# R-spondin 2 promotes acetylcholine receptor clustering at the neuromuscular junction via Lgr5

**DOI:** 10.1038/srep28512

**Published:** 2016-06-22

**Authors:** Hiroaki Nakashima, Bisei Ohkawara, Shinsuke Ishigaki, Takayasu Fukudome, Kenyu Ito, Mikito Tsushima, Hiroyuki Konishi, Tatsuya Okuno, Toshiro Yoshimura, Mikako Ito, Akio Masuda, Gen Sobue, Hiroshi Kiyama, Naoki Ishiguro, Kinji Ohno

**Affiliations:** 1 Division of Neurogenetics, Center for Neurological Diseases and Cancer, Nagoya University Graduate School of Medicine, Nagoya, Japan; 2 Department of Orthopedic Surgery, Nagoya University Graduate School of Medicine, Nagoya, Japan; 3 Department of Neurology, Nagoya University Graduate School of Medicine, Nagoya, Japan; 4 Department of Neurology, Nagasaki Kawatana Medical Center, Nagasaki, Japan; 5 Department of Functional Anatomy and Neuroscience, Nagoya University Graduate School of Medicine, Nagoya, Japan; 6 Department of Occupational Therapy, Nagasaki University School of Health Sciences, Nagasaki, Japan

## Abstract

At the neuromuscular junction (NMJ), acetylcholine receptor (AChR) clustering is mediated by spinal motor neuron (SMN)-derived agrin and its receptors on the muscle, the low-density lipoprotein receptor-related protein 4 (LRP4) and muscle-specific receptor tyrosine kinase (MuSK). Additionally, AChR clustering is mediated by the components of the Wnt pathway. Laser capture microdissection of SMNs revealed that a secreted activator of Wnt signaling, R-spondin 2 (Rspo2), is highly expressed in SMNs. We found that Rspo2 is enriched at the NMJ, and that Rspo2 induces MuSK phosphorylation and AChR clustering. Rspo2 requires Wnt ligands, but not agrin, for promoting AChR clustering in cultured myotubes. Leucine-rich repeat-containing G-protein coupled receptor 5 (Lgr5), an Rspo2 receptor, is also accumulated at the NMJ, and is associated with MuSK via LRP4. Lgr5 is required for Rspo2-mediated AChR clustering in myotubes. In *Rspo2*-knockout mice, the number and density of AChRs at the NMJ are reduced. The *Rspo2*-knockout diaphragm has an altered ultrastructure with widened synaptic clefts and sparse synaptic vesicles. Frequency of miniature endplate currents is markedly reduced in *Rspo2*-knockout mice. To conclude, we demonstrate that Rspo2 and its receptor Lgr5 are Wnt-dependent and agrin-independent regulators of AChR clustering at the NMJ.

The neuromuscular junction (NMJ) is the synapse that is formed between a spinal motor neuron (SMN) and the skeletal muscle. Contraction of the skeletal muscle is controlled by the neurotransmitter acetylcholine (ACh), which is released from the motor nerve terminal. To achieve efficient neuromuscular transmission, acetylcholine receptors (AChRs) at the muscle endplate must be densely clustered within the postsynaptic membrane of the NMJ in direct apposition to the presynaptic active zones^1^,^2^,^3^. AChR clustering is mediated by the SMN-derived agrin^4^,^5^,^6^. Agrin binds to the low-density lipoprotein receptor-related protein 4 (LRP4) on the postsynaptic membrane and it phosphorylates the muscle-specific receptor tyrosine kinase (MuSK)^7^,^8^, which leads to the facilitation of AChR clustering through a scaffold composed of the subsynaptic structural protein rapsyn^9^,^10^. At an early stage of development, prepatterned AChR clusters around the center of muscle fibers are induced by the components of Wnt signaling^11^,^12^. Agrin has no homologue in Drosophila, and the Wnt homologue wingless solely induces the growth of the larval NMJ^13^. In zebrafish and mice, knockout/knockdown of Wnt ligands^14^,^15^,^16^, dishevelled (Dvl)^15^,^16^ and β-catenin^17^,^18^,^19^, demonstrate that these molecules regulate the positioning of AChR clusters. β-catenin promotes AChR clustering by directly associating with AChR^20^. Similarly, other intracellular Wnt signaling components, such as Dvl and casein kinase 2, regulate AChR clustering in cultured myotubes^21^,^22^. In general, the strictly regulated expression of secreted antagonists including Dickkopf (Dkk) and secreted frizzled-related protein (Sfrps) and agonists such as R-spondins (Rspos) and Norrin enable the fine spatiotemporal modulation of Wnt signaling^23^,^24^. For example, the Wnt antagonist Sfrp1 is induced by denervation at rat NMJs, and it suppresses Wnt signaling to disperse AChR clusters^24^. However, no agonist of the Wnt signaling pathway is known to work on the NMJ formation.

Four secreted Rspos have unique Wnt-enhancing abilities, and their expression patterns are tightly regulated in a tissue- and developmental-stage–specific manner. A cell surface binding assay^25^, a co-immunoprecipitation assay^26^, and a high resolution crystal structure^27^ showed that Rspo2 directly binds to the leucine-rich-repeat–containing G-protein coupled receptor (Lgr) 4 and 5; its binding activates the Wnt receptors (Frizzled and LRP6) on the cell membrane^28^,^29^,^30^,^31^,^32^,^33^. Rspo2 is involved in the development of several tissues. Rspo2 is required for the development of the larynx, trachea, and lung^34^. In addition, Rspo2 can regulate early muscle differentiation^35^. Expression of Rspo family proteins in the brain was previously reported using *in situ* hybridization^36^; however, the function of Rspo proteins in the neural and neuromuscular networks remains to be elucidated. Here we report that a secreted protein, Rspo2, is highly expressed in the SMNs and binds to its receptor, Leucine-rich repeat-containing G-protein coupled receptor 5 (Lgr5), at the NMJ. Rspo2 enhances the LRP4/MuSK signaling via Lgr5 in an agrin-independent manner and promotes AChR clustering. In addition, the loss of *Rspo2* in mice compromises AChR clustering, the ultrastructure of the NMJ, and neuromuscular signal transduction.

## Results

### Rspo2 is a highly expressed Wnt-related gene in SMNs

**Figure 1:**
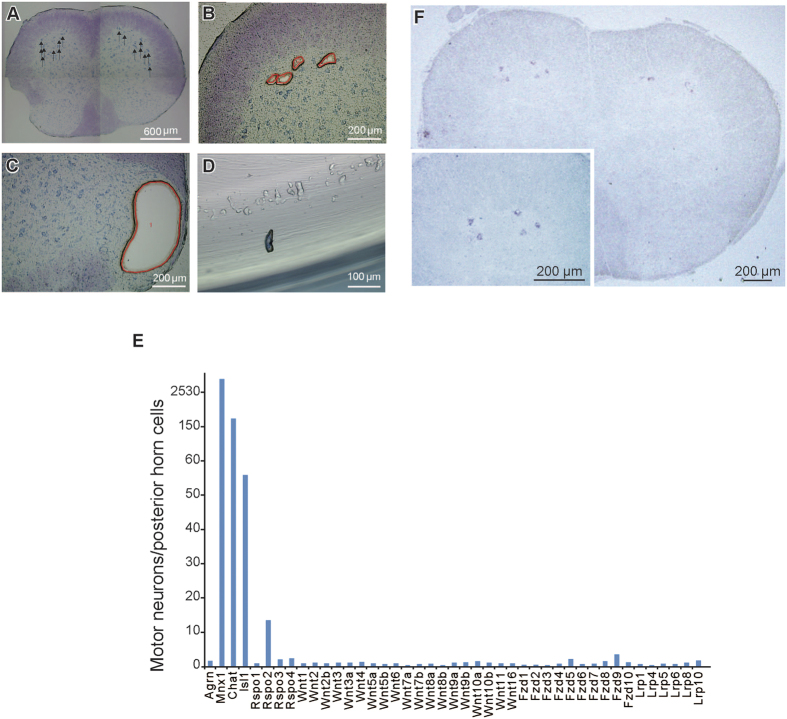
R-spondin 2 (Rspo2) is highly expressed in laser capture microdissection-harvested spinal motor neurons (SMNs) of the mouse spinal cord. (**A**) Toluidine blue-stained section of the cervical spinal cord of a 6-week-old C57BL6/J mouse before laser capture microdissection. Arrows indicate SMNs to be dissected. (**B**) The left anterior horn region (enlarged from **A**) after the dissection of SMNs. Orange lines mark the traces of the laser beam. (**C**) The right posterior horn region (enlarged from **A**) after the dissection of posterior horn cells. Orange line marks the trace of the laser beam. (**D**) A representative dissected SMN. (**E**) The ratio of mRNA expressions in SMNs and posterior horn cells of *Agrn, Chat, Isl1, Mnx1*, and Wnt-related genes (*Rspo, Wnt, Fzd*, and *Lrp* genes) according to the Affymetrix microarray data. *Agrn, Chat, Isl1, Mnx1, Fzd, and Lrp* encode agrin, choline acetyltransferase, islet-1, HB9, frizzled, and low-density lipoprotein receptor-related protein, respectively. (**F**) *In situ* hybridization of *Rspo2* in the cervical spinal cord of a 6-week-old C57BL6/J mouse.

To screen for proteins that could potentially participate in NMJ formation, we harvested ~3,000 SMNs from three 6-week-old C57BL6/J mice using laser capture microdissection (Fig. 1B). As a control, we harvested cells from the posterior horn cells (Fig. 1C). We analyzed gene expression using the Affymetrix Exon 1.0 ST array (Fig. 1E) and RNA-sequencing (RNA-seq) (Supplementary Fig. S1A). We found that the expression levels of 164 genes were more than 10 times higher in SMNs than in the posterior horn cells (Supplementary Table S1). *Agrn* encoding agrin, *Mnx1* encoding HB9, *Chat* encoding choline acetyltransferase, and *Isl1* encoding islet1 are commonly used markers for SMNs. SMN-specificity of *Rspo2* expression was the highest among the 39 Wnt-related genes, although it was lower than those of *Mnx1*, *Chat*, and *Isl1* (Fig. 1E). Similar to the results of the microarray, RNA-seq analysis showed a 14.9-fold higher expression of *Rspo2* in SMNs than in the posterior horn cells (Supplementary Fig. S1A). We also confirmed specific expression of *Rspo2* in SMNs by *in situ* hybridization (Fig. 1F) in the spinal cord, which revealed a similar hybridization pattern to that in the Allen Mouse Brain Atlas (http://mouse.brain-map.org/). In addition, Rspo2 and choline acetyltransferase (ChAT) were co-expressed in anterior horn cells by immunohistochemistry (Supplementary Fig. S1B).

### Rspo2 enhances LRP4/MuSK signaling and induces AChR clustering via Lgr5 in a Wnt-dependent and agrin-independent manner

**Figure 2:**
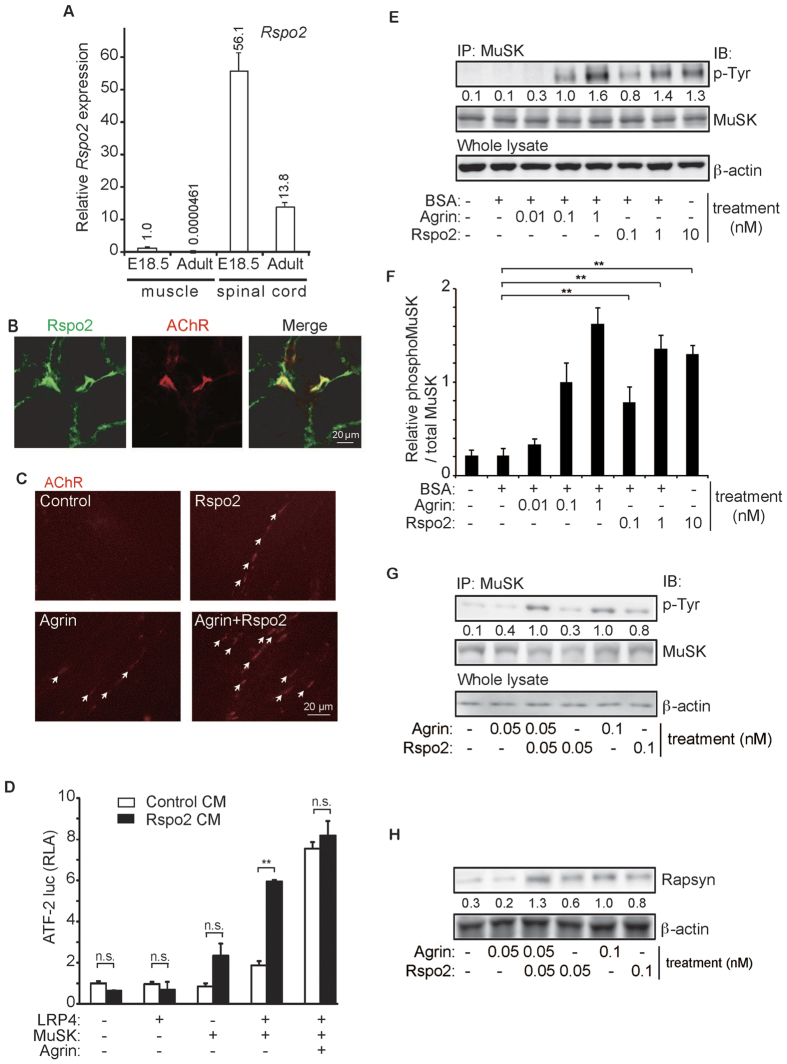
Rspo2 is enriched at the NMJ and activates MuSK to induce AChR clustering. (**A**) Rspo2 expression in the diaphragm and spinal cord normalized by *Gapdh* and also to E18.5 diaphragm. Mean and SD (*n* = 3) are indicated. (**B**) Rspo2 immunostaining and α-bungarotoxin staining for AChR at the NMJ of the tibialis anterior muscle (cross section). (**C**) AChR clusters visualized with α-bungarotoxin (red). C2C12 myotubes were cultured with 0.05 nM agrin and/or 0.05 nM Rspo2. Arrows point to the AChR clusters with an axis length of 4 μm or more. Blinded morphometric analysis is shown in Supplementary Fig. S2A. (**D**) ATF2-luciferase reporter assay to quantify agrin (10 ng/ml)- and Rspo2 (100 ng/ml)-mediated activation of MuSK signaling in transfected HEK293 cells. Relative luciferase activities (RLA) are normalized to that with empty vectors. Mean and SD are indicated (*n* = 3). **p < 0.01 by *t*-test. n.s., no significant difference. (**E**) Agrin- and Rspo2-mediated MuSK phosphorylation in C2C12 myotubes. Phosphorylated MuSK was immunoprecipitated (IP) and immunoblotted (IB) with indicated antibodies. (**F**) The ratio of phosphorylated (phosphoMuSK) and total MuSK was normalized to that with 0.1 nM agrin. Mean and SD (*n* = 3) are indicated. **p < 0.01 by *t*-test. The mean is also indicated in (**E**). BSA was added to control the amount of total proteins. (**G**) Additive effect of 0.05 nM agrin and 0.05 nM Rspo2 on MuSK phosphorylation in C2C12 myotubes. Phosphorylated MuSK was detected as in (**E**). Band intensities were normalized to that with 0.1 nM agrin (Supplementary Fig. S2B). The mean intensity is also is indicated below the blot. BSA was added as in (**E**). (**H**) Agrin- and Rspo2-mediated expression of rapsyn in C2C12 myotubes. Band intensities were normalized as in (**G**) (Supplementary Fig. S2C). The mean value is also indicated below the blot.

We first compared the expression of Rspo2 in the skeletal muscles and the spinal cord. Gene expression level of *Rspo2* was 56 times higher in the spinal cord than that in the skeletal muscles at embryonic day 18.5 (E18.5) (Fig. 2A), and was ~300,000 times higher in adults (Fig. 2B). Nevertheless, Rspo2 was enriched at the NMJs together with AChR clusters in adult skeletal muscles, and also along the muscle plasma membrane to a lesser extent (Fig. 2B and Supplementary Fig. S1C). The enrichment of the Rspo2 at the NMJs prompted us to investigate the function of Rspo2 at the NMJs.

Wnt ligands induce MuSK phosphorylation and subsequent AChR clustering *in vitro* and *in vivo*^15^,^16^,^37^. Therefore, we examined the effect of Rspo2 on AChR clustering in cultured myotubes in C2C12 myotubes, and found that exogenous Rspo2 enhanced AChR clustering (Fig. 2C and Supplementary Fig. S2A). We next evaluated the effects of Rspo2 on MuSK activation using a c-Jun N-terminal kinase (JNK)-responsive activating transcription factor 2 (ATF-2) luciferase reporter (ATF2-luc)^38^ (Fig. 2D). As previously reported^39^,^40^, agrin activated ATF2-luc in MuSK/LRP4-cotransfected HEK293 cells. Addition of Rspo2-conditioned medium (CM) activated ATF2-luc in MuSK-transfected HEK293 cells, and more prominent activation was observed in MuSK/LRP4-cotransfected HEK293 cells. Purified recombinant Rspo2 protein similarly enhanced the phosphorylation of MuSK in a dose-dependent manner in C2C12 cells (Fig. 2E,F). In addition, Rspo2 had an additive effect on agrin-mediated AChR clustering (Fig. 2C and Supplementary Fig. S2A), MuSK phosphorylation (Fig. 2G and Supplementary Fig. S2B), and Rapsyn expression (Fig. 2H and Supplementary Fig. S2C).

**Figure 3:**
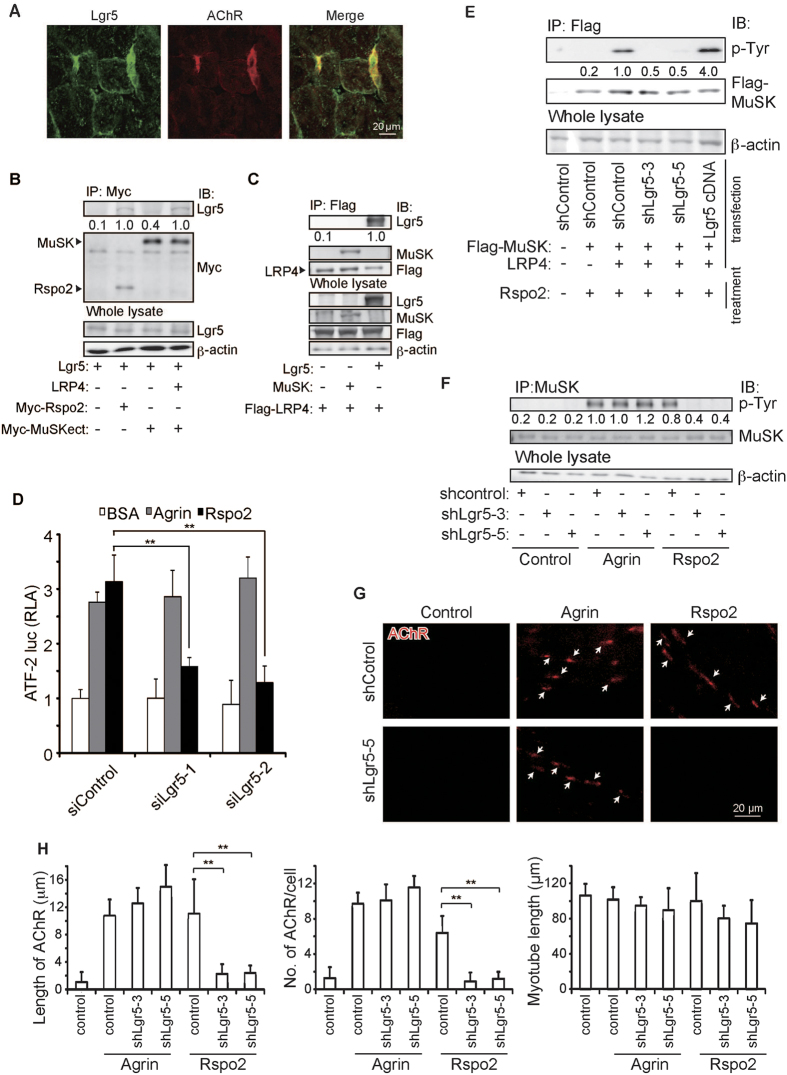
The Rspo2/Lgr5 complex induces activation and phosphorylation of MuSK and AChR clustering. (**A**) Lgr5 immunostaining and α-bungarotoxin staining of the NMJs as in Fig. 2B. (**B,C**) Lgr5 was co-immunoprecipitated with anti-myc (B) or anti-Flag (C) antibody in HEK293 cells. The normalized ratio of co-immunoprecipitated Lgr5 to total Lgr5 is shown in Supplementary Fig. S3A and S3B, and the mean value is indicated below the blot. (**D**) ATF2-luciferase reporter assays as in Fig. 2D in siRNA-transfected HEK293 cells. RLA are normalized to that of BSA with siControl. Mean and SD are indicated (**p < 0.01 by *t*-test, *n* = 3). Efficiency of siLgr5 and the rescue experiment is shown in Supplementary Figs S3B and S3C, respectively. (**E**) The effects of Lgr5 on MuSK phosphorylation in L cells treated with 0.1 nM Rspo2. Total Flag-MuSK was immunoprecipitated (IP), and immunoblotted (IB) with indicated antibodies. The ratio of phosphorylated and total Flag-MuSK was normalized to that of the control knockdown (shControl) in lane 3. Quantification is shown in Supplementary Fig. S3F, and the mean value is indicated below the blot. Efficiency of shLgr5 in L cells is indicated in Supplementary Fig. S3E. (**F**) C2C12 myoblasts were infected with lentivirus expressing shControl or shLgr5. After differentiation into myotubes, 0.1 nM of BSA (Control), agrin, or Rspo2 was added. Phosphorylated MuSK was immunoprecipitated (IP) and immunoblotted (IB) with indicated antibodies. The band intensities were normalized to that of cells treated with 0.1 nM agrin and shControl. Quantification of phosphorylated MuSK is shown in Supplementary Fig. S3H, and the mean value is indicted below the blot. Efficiency of shLgr5 in C2C12 myotubes is indicated in Supplementary Fig. S3G. (**G,H**) C2C12 myoblasts were infected with lentivirus expressing shControl or shLgr5. After differentiation into myotubes, 0.1 nM of BSA (Control), agrin, or Rspo2 was added. AChR cluster was visualized with α-bungarotoxin (red). (**F**) Arrows point to AChR clusters in representative images. (**G**) Blinded morphometric analysis. Mean and SD are indicated (**p < 0.01 by *t*-test, *n* = 3). Instead of purified recombinant Rspo2, we also used Rspo2-containing conditioned medium, and showed the results in Supplementary Fig. S2D.

Lgr5 is a receptor of Rspo proteins in the intestinal crypts and hair follicles^26^,^31^. We first observed that Lgr5 was colocalized with AChR at the NMJ in wild-type mice (Fig. 3A). Colocalization of Rspo2 and Lgr5 at the NMJs suggested that Lgr5 might be a receptor for Rspo2 at the NMJs. Therefore, we performed immunoprecipitation assays to examine whether Lgr5 was able to associate with MuSK. As previously reported^26^,^27^, Lgr5 was co-immunoprecipitated with Rspo2 (Fig. 3B). We found that Lgr5 was not co-immunoprecipitated with MuSK (Fig. 3B and Supplementary Fig. S3A). However, coexpression of LRP4 enabled co-immunoprecipitation of Lgr5 with MuSK (Fig. 3B and Supplementary Fig. S3A). As LRP4 was able to co-immunoprecipitate Lgr5 and MuSK (Fig. 3C and Supplementary Fig. S3B), Lgr5 binds to MuSK via LRP4 and not directly. We next examined the effects of Lgr5 on Rspo2-mediated MuSK activation in HEK293, L, and C2C12 cells. First, in MuSK/LRP4-cotransfected HEK293 cells, knockdown of Lgr5 (Supplementary Fig. S3C) markedly suppressed Rspo2-mediated, but not agrin-mediated, activation of ATF2-luc (Fig. 3D). Second, in L cells, we examined the phosphorylation of MuSK using a lentivirus that expressed an inducible shRNA against *Lgr5*. Knockdown of Lgr5 in MuSK/LRP4-cotransfected L cells (Supplementary Fig. S3E) suppressed Rspo2-mediated phosphorylation of MuSK (Fig. 3E and Supplementary Fig. S3F). In contrast, the overexpression of Lgr5 enhanced Rspo2-mediated phosphorylation of MuSK (Fig. 3E and Supplementary Fig. S3F). Third, in C2C12 myotubes, knockdown of Lgr5 (Supplementary Fig. S3G) suppressed Rspo2-mediated, but not agrin-mediated, phosphorylation of MuSK (Fig. 3F and Supplementary Fig. S3H). In addition, Rspo2 enhanced the expression of membrane-bound LRP4, but not of membrane-bound MuSK, in the mouse diaphragm (Supplementary Fig. S4A–C) and in C2C12 myotubes (Supplementary Fig. S4D,E).

We next examined if the positive effect of Rspo2 on MuSK phosphorylation was Wnt-dependent. IWP-2, an inhibitor of Wnt secretion, abrogated the Rspo2-mediated phosphorylation of MuSK in C2C12 myotubes (Supplementary Fig. S3I). Thus, Wnt ligands are necessary for Rspo2-mediated phosphorylation of MuSK.

We also examined the effects of Lgr5 on Rspo2-mediated AChR clustering in myotubes. We introduced a lentivirus expressing an inducible shRNA against *Lgr5* in C2C12 myotubes. In *Lgr5*-deficient C2C12 myotubes, agrin increased the length and number of AChR clusters, whereas Rspo2 could not induce the formation of AChR clusters (Fig. 3G,H and Supplementary Fig. S2D). These results suggest that Lgr5 is a receptor for Rspo2, and is required for phosphorylation of MuSK and AChR clustering mediated by Rspo2, but not by agrin.

### Loss of Rspo2 compromises AChR clustering as well as the ultrastructure and signal transduction at the NMJs in *Rspo2*-deficient (−/−) mice at E18.5

**Figure 4:**
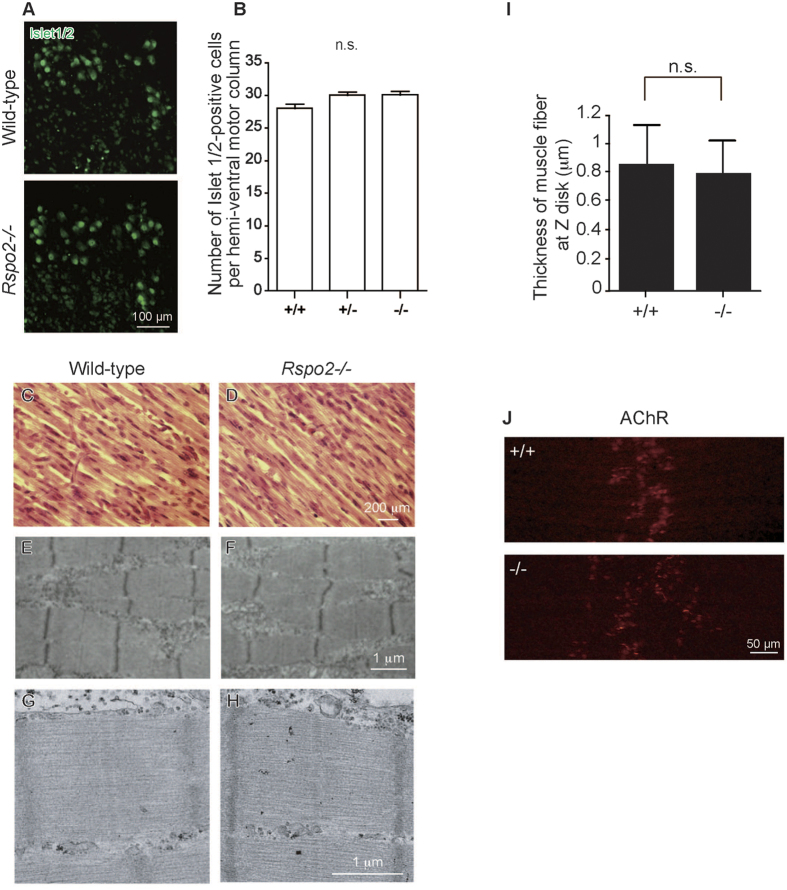
Lack of R-spondin 2 (Rspo2) in mice has minimal effects on spinal motor neuron (SMN) survival and muscle differentiation, but has a significant effect on acetylcholine receptor (AChR) clusters in the left diaphragm. (**A**) Immunostaining for islet1/2 expressed in the SMNs of the spinal cord (C3-C6) at embryonic day (**E**) 18.5. (**B**) The number of islet1/2-positive SMNs in wild-type (+/+), heterozygous *Rspo2*-knockout (+/−), and homozygous *Rspo2*-knockout (−/−) mice. Bars indicate the mean and standard error of mean (SE) (*n* > 90). No statistically significant differences (n.s.) were observed with one-way ANOVA. (**C,D**) Hematoxylin and eosin staining of the tibialis anterior muscle of mice at E18.5. (**E–H**) Representative electron micrographs of the diaphragms at E18.5 at different magnifications. (**I**) Thickness of muscle fibers at the Z disk in the left diaphragms of wild-type (+/+) and *Rspo2*-knockout (−/−) mice. Five to seven electron micrographs were analyzed in each mouse. (**J**) Surface views of the left diaphragms harvested from wild-type (+/+) and *Rspo2*-knockout (−/−) mice at E18.5. AChR was stained with Alexa546-conjugated α-bungarotoxin (red). The widths of the AChR bands of wild-type and *Rspo2*-kockout diaphragms were 143.34 ± 3.73 μm and 221.85 ± 6.52 μm, respectively (*p* < 0.0001 by Student’s *t*-test, *n* = 5).

To evaluate the function of Rspo2 *in vivo*, we examined *Rspo2*−/− mice. *Rspo2*−/− mice die shortly after birth due to respiratory distress^34^,^41^,^42^. To elucidate the function of Rspo2 in the spinal cord and skeletal muscle during embryonic development, we first counted the number of islet1/2-positive SMNs in the C3-C6 spinal segments of E18.5 mice. The number of SMNs did not change in *Rspo2*−/− mice, indicating that Rspo2 is not required for the development and survival of SMNs (Fig. 4A,B). Next, we examined muscle differentiation in the *Rspo2*−/− diaphragm at E18.5. Although Rspo2 was previously reported to enhance early myogenic differentiation in cultured C2C12 cells^35^, the microscopic structure (Fig. 4C,D) and ultrastructure (Fig. 4E–H) of muscle fibers in *Rspo2*−/− mice were similar to those in wild-type mice at E18.5. Quantification of the thickness of muscle fibers at the Z disk also revealed that wild-type and *Rspo2*−/− mice had a similar muscle fiber thickness (Fig. 4I). These results suggest that *Rspo2* deficiency does not affect muscle differentiation in the diaphragm at E18.5.

**Figure 5:**
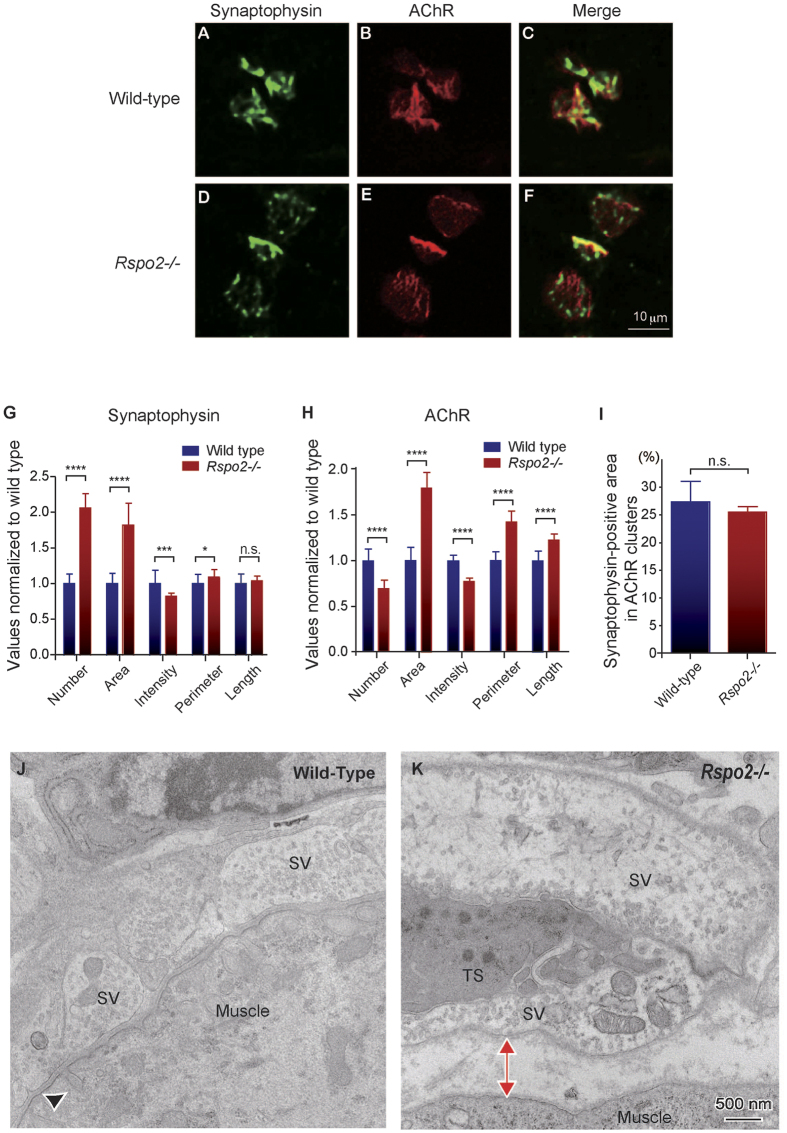
Apposition of nerve terminals and muscle endplates is compromised in the E18.5 diaphragm of R-spondin 2 (*Rspo2)*−/− mice. (**A–F**) Representative confocal images of the left diaphragm at E18.5 labeled with an anti-synaptophysin antibody and α-bungarotoxin to visualize the nerve terminals and acetylcholine receptors (AChRs), respectively. Endplates of the wild-type muscles were mostly ovoid-shaped (**B**), whereas the endplates of *Rspo2*−/− muscles were large, round, and heterogeneously stained (**E**). (**G–I**) Blinded morphometric analysis of synaptophysin (**G**) and AChR (**H**) in the AChR clusters revealed that NMJ areas were markedly enlarged at E18.5. Numbers of synaptophysin-positive clusters (**G**) and AChR-positive clusters (**H**) are shown. (**I**) The ratio is calculated by dividing the synaptophysin-positive area by the AChR-positive area. Note that not all AChR-positive (red) pixels were synaptophysin-positive (green) in each AChR cluster. Mean and standard deviation (SD; *n* = 6) are indicated. ****p < 0.001, ***p < 0.005, and *p < 0.05 by *t*-test. n.s., no significant difference. (**J,K**) Representative electron micrographs of the neuromuscular junctions (NMJs) in the diaphragm of wild-type and *Rspo2*−/− mice at E18.5. The red two-headed arrow indicates a widened synaptic cleft. The closed arrowhead at wild-type endplate points to a postsynaptic fold. In the *Rspo2*−/− mice, synaptic vesicles were larger and sparser than those in wild-type mice. SV, synaptic vesicles; TS, terminal Schwann cell. Low magnification images are shown in Supplementary Fig. S6A. Blinded morphometric measurements are shown in Table 1.

We then investigated the NMJs of *Rspo2*−/− mice. At E14.5, the sizes of AChR clusters were slightly larger in the left diaphragm of *Rspo2*−/− mice compared to those in wild-type mice (Supplementary Fig. S5A and B). At E18.5, AChR clusters in the left diaphragm formed a broader band in *Rspo2*−/− mice compared to those in wild-type mice (Fig. 4J). In addition, at E18.5. blinded morphometric analysis of AChR clusters and the nerve terminals (Fig. 5A–F) revealed that the synaptophysin-positive nerve terminal area (Fig. 5G) and the AChR-positive endplate area (Fig. 5H) were both approximately two-fold larger in *Rspo2*−/− mice than wild-type mice. As the ratio of the synaptophysin-positive area within each AChR cluster remained unchanged (Fig. 5I), the nerve terminals was likely to be properly appositioned to AChR clusters in *Rspo2*−/− mice. The number of AChR clusters was decreased (Fig. 5H), which likely represents that AChR clusters became expanded and fused each other to make a fewer number of weakly stained AChR clusters. In contrast, the number of synaptophysin-positive areas was not likely to be fused, and its number was increased in *Rspo2*−/− mice (Fig. 5G). The signal intensities of AChR and synaptophysin were reduced in *Rspo2*−/− mice (Fig. 5G,H). In accordance with the abnormal AChR clustering, the lengths of the second and third axonal branches were increased in *Rspo2*−/− mice at E18.5 (Supplementary Fig. S5C, D). Similarly, the number of the second and third axonal branches were decreased and increased, respectively (Supplementary Fig. S5C,E). The ratio of the NMJs terminated by an axon in *Rspo2*−/− mice at E18.5 was similar to that of wild-type mice (Supplementary Fig. S5F,G).

In addition to the abnormal AChR clustering, the ultrastructure of the NMJs in *Rspo2*−/− diaphragms at E18.5 was different from that in wild-type diaphragms (Fig. 5J,K and Supplementary Fig. S6). Blinded morphometric analysis revealed that the synaptic clefts were wider in *Rspo2*−/− mice (Table 1). In addition, the density and size of synaptic vesicles were lower and larger in *Rspo2*−/− mice, respectively. The number of active zones per synapse was also lower in *Rspo2*−/− mice. In concordance with abnormality at the pre-synaptic regions, the post-synaptic regions of *Rspo2*−/− NMJs showed significantly fewer junctional folds.

To evaluate neuromuscular signal transduction in *Rspo2*−/− mice, we analyzed the miniature endplate potentials (MEPPs) of the left diaphragm and the compound muscle action potentials (CMAPs) of the tibialis anterior muscle. Amplitudes of MEPPs were slightly higher, and the frequencies of MEPPs were markedly lower in *Rspo2*−/− mice (Table 2). The increased MEPP amplitude may be caused by the large synaptic vesicles stated above (Fig. 5J,K, Supplementary Fig. S6, and Table 1). Similarly, the markedly decreased MEPP frequency may be caused by the widened synaptic cleft and the reduced number of active zones. Defective neuromuscular signal transmission was also confirmed on the basis of the abnormally decreased CMAP amplitude measured in the tibialis anterior muscle after the repetitive stimulation of the sciatic nerve (Table 2). A similar phenomenon is commonly observed in myasthenia gravis and in congenital myasthenic syndromes, in which the NMJ signal transmission is compromised^43^. These results suggest that Rspo2 plays an essential role in AChR clustering and the associated signal transmission at the NMJs.

## Discussion

**Figure 6:**
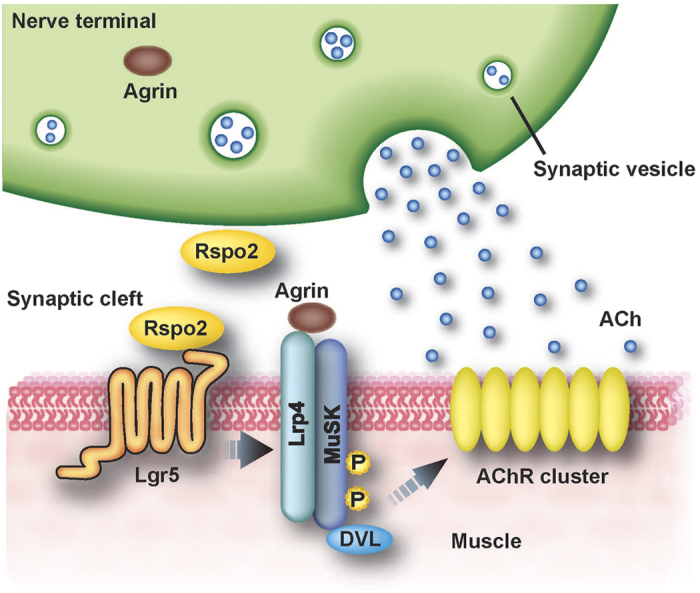
Schematic image showing the R-spondin 2 (Rspo2)-mediated acetylcholine receptor (AChR) clustering at the neuromuscular junction (NMJ). Rspo2 binds to leucine-rich repeat-containing G-protein coupled receptor 5 (Lgr5) on the endplate and phosphorylates muscle-specific receptor tyrosine kinase (MuSK) to induce AChR clustering in an agrin-independent manner.

We found that Rspo2 is a Wnt-dependent and agrin-independent regulator of MuSK phosphorylation and AChR clustering at the NMJs (Fig. 6). Rspo2 is highly expressed in SMNs; Rspo2 at the NMJs is likely to be derived from SMNs. Lgr5 is expressed at the NMJs and is a receptor for Rspo2.

Rspo2 is highly expressed in the SMNs of the spinal cord (Fig. 1F). Although SMN-specificity of *Rspo2* expression is less than those of SMN marker genes (*Chat, Isl1*, and *Mnx1*) (Fig. 1E), the expression level of *Rspo2* in SMNs is as high as that *Agrn* and more than those of *Mnx1* and *Isl1* by RNA-seq (Supplementary Fig. S1A). On the other hand, the specificity of *Rspo2* expression in SMNs is the highest among the Wnt-related genes including various Wnt, Fzd, Rspo, and Lrp genes (Fig. 1E). Gene expression of *Rspo2* in SMNs is much higher than that in skeletal muscles at E18.5 and in adults (Fig. 2A). Nevertheless, Rspo2 protein is enriched at the NMJs (Fig. 2B). According to the Allen Brain Atlas (http://mouse.brain-map.org/), GenePaint (http://www.genepaint.org/), and Wnt Gene Expression Patterns in the Embryo (http://www.tcd.ie/Zoology/research/WntPathway/wnt.php), Wnt ligands are expressed in both SMNs and skeletal muscles during synaptogenesis. Notwithstanding the broad expression of Wnt ligands, the mechanism of NMJ-specific Wnt signaling pathway activation has not been dissected yet. Rspo proteins are not expressed in primary Schwann cells isolated from mouse embryos^44^. Global expression analyses of mouse NMJs obtained with laser capture microdissection demonstrated that *Rspo2* is expressed in both the subsynaptic and extrajunctional nuclei^45^,^46^,^47^,^48^, although its expression level in skeletal muscles is much lower than that in SMNs. Thus, Rspo2 derived from SMNs might enable the NMJ-specific activation of Wnt signaling. However, spatiotemporal conditional knockouts of *Rspo2* will be required to verify the origin of Rspo2 that mediates the Wnt-dependent AChR clustering.

In cultured cells, the effects of Rspo2 on AChR clustering (Fig. 2C), ATF2 activation (Fig. 2D), and MuSK phosphorylation (Fig. 2E–G) are similar to those of agrin^4^,^5^,^6^. Agrin has two splicing isoforms: the muscle and the neuronal types^4^. Neuronal-type agrin, which has two alternatively spliced exons, is able to drive AChR clustering, whereas muscle-type agrin lacks these critical exons, and cannot drive AChR clustering. According to gene annotation databases (RefSeq, ENSEMBL, UCSC, and GENCODE), mouse *Rspo2* is not alternatively spliced in its coding region. We also confirmed lack of alternative splicing isoforms in mouse *Rspo2* by RT-PCR and RNA-seq (data not shown). Thus, unlike the expression of agrin, the expression of Rspo2 is likely to be regulated in a spatiotemporal manner and not by alternative splicing.

In cultured cells, Rspo2 binds to Lgr5 (Fig. 3B). Lgr5 (Fig. 3A) and Rspo2 (Fig. 2B) are enriched at the NMJs in wild-type mice. In addition, the positive effects of Rspo2 on MuSK phosphorylation (Fig. 3E,F) and AChR clustering (Fig. 3G,H) were dependent on Lgr5. We^39^ and others^49^ previously reported that Rspo proteins do not bind to Frizzled receptors carrying the frizzled-like domain, which is also present in MuSK. Instead, Rspo2 specifically binds to Lgr4 and Lgr5^25^,^26^,^31^. Northern blot analysis^50^, the H-ANGEL database (http://h-invitational.jp/)^51^, and the Fantom 5 database (http://fantom.gsc.riken.jp/zenbu/)^52^ indicate that the skeletal muscle exclusively expresses Lgr5, but not Lgr4 or Lgr6. Interestingly, *Lgr5*-deficient mice show craniofacial malformations and perinatal lethality^53^, similarly to our observations in *Rspo2*-deficient mice. Further studies of *Lgr5*-deficient mice might reveal the *in vivo* functions of Lgr5 at the NMJs in association with Rspo2.

Lgr5 is able to associate with MuSK in an LRP4-dependent manner (Fig. 3B,C). Additionally, Rspo2 increases the amount of membrane-bound LRP4 (Supplementary Fig. S4). In intestinal stem cells, Binding of Rspo proteins to Lgr proteins stabilize Fzd and Lrp molecules by the inhibition of their ubiquitination^31^. Therefore, Rspo2 at the NMJs may similarly stabilize LRP4, which enhances the effect of Rspo2 by facilitating binding of Lgr5 and MuSK, and the effect of agrin by increasing the number of LRP4/MuSK co-receptor. Our findings provide the first evidence that a secreted Wnt signaling agonist, Rspo2, modulates AChR clustering together with Wnt proteins, Lgr5, LRP4, and MuSK.

The diaphragm of *Rspo2*−/− mice show an expanded area and a decreased intensity of AChR clusters at E18.5 (Fig. 5A–H). Additionally, the synaptic clefts are wider in *Rspo2*−/− mice (Fig. 5J,K and Table 1). Widened synaptic clefts have already been documented in *Agrn*−/− mice^54^, but not in other knockout mice deficient for NMJ-constituent molecules such as β2-laminin (*Lamb2*)^55^, AChR ε subunit (*Chrne*)^56^, utrophin (*Utrn*)^57^, neural cell adhesion molecule (*Ncam*)^58^, tenascin C (*Tnc*)^58^, fibroblast growth factor 5 (*Fgf5*)^58^, and choline acetyltransferase (*Chat*)^59^ (Table 3). As the width of the synaptic cleft is not always described precisely in these reports, the specificity of widened synaptic clefts observed in *Rspo2*−/− and *Agrin*−/− mice remains unknown. Furthermore, the density and size of synaptic vesicles are lower and larger in *Rspo2*−/− mice, respectively (Fig. 5J,K and Supplementary Fig. S6). MEPP amplitudes are slightly increased, and MEPP frequencies are markedly decreased in *Rspo2*−/− mice, which is consistent with the larger size and lower density of synaptic vesicles in electron micrographs (Tables 1 and 2). The markedly decreased MEPP frequencies may be associated with the widened synaptic clefts. In *Chat*−/− mice, the density of synaptic vesicles is reduced to approximately 80% of that in wild-type^59^. Reduction in the density of synaptic vesicles to approximately 60% of that in wild-type mice in *Rspo2*−/− mice (Table 1) suggests that Rspo2 deficiency may compromise the recycling of synaptic vesicles. In quiescent nerve terminals, the size of synaptic vesicles become large, which might be due to a low releasing rate of ACh^60^,^61^. Thus, large synaptic vesicles in *Rspo2*−/− mice may be caused by reduced synaptic release, which is suggested by the markedly reduced MEPP frequency. We also observed that the number of active zones is reduced in *Rspo2*−/− mice (Table 1). In *Agrn*−/−54 and *Ctnnb1*−/− mice^18^, the number of active zones is also reduced (Table 3). Similarly, in *Chrne*−/−56 and *Ncam*−/− mice^58^, the number of junctional folds is reduced. In contrast, in *Lamb2*−/− mice, the number of active zones is preserved^55^. Thus, the reduced number of active zones or junctional folds might be a hallmark of defective neuromuscular signal transmission in most, but not all, mice deficient for NMJ-constituent molecules. We unexpectedly observed the increased size of AChR clusters in *Rspo2*−/− mice (Fig. 5H). Interestingly, the increased size of AChR clusters is also present in knockout mice lacking *Wnt4*^15^ and *Ctnnb1* encoding β-catenin^18^ (Table 3). In contrast, this phenotype has not been documented in *Agrn*−/− mice^54^. The increased size of AChR clusters may be a unique feature of mice deficient for Wnt-related molecules, but the underlying mechanism remains unknown. We propose that Rspo2 might enable the NMJ-specific activation of Wnt signaling to induce AChR clustering.

## Materials and Methods

### Laser capture microdissection, micro array analysis, and RNA-seq

All experiments with mice were approved by the Animal Care and Use Committee of the Nagoya University Graduate School of Medicine, and were performed in accordance with the relevant guidelines. The cervical spinal cords of 6-week-old C57BL/6J male mice were dissected without fixation under deep anesthesia. Frozen sections (10 μm) were prepared and stained with 0.02% toluidine blue, and SMNs were clipped out of the sections using laser microdissection (LMD7000, Leica) according to the manufacturer’s protocol. We collected ~3000 cells that were larger than 20 μm in diameter from the anterior horn area of three mice, and ~20 dorsal horn areas were collected from three mice. RNA was extracted using the RNeasy Micro kit (Qiagen). RNA quality was evaluated with the Agilent Model 2100 Bioanalyzer (Agilent Technologies). RNA was subjected to linear amplification using the Ovation Pico WTA System (NuGEN Technologies). For the microarray analysis, fluorescent cDNA probes were synthesized using the FL-Ovation cDNA Biotin Module V2 (NuGEN Technologies) and WT-Ovation Exon Module (NuGEN Technologies). Gene expression profiles were analyzed using the Affymetrix Mouse Exon 1.0 ST Array according to the manufacturer’s protocol. We also sequenced 75 base pairs of each tag in a single direction using HiSeq 2000 (Illumina). SMNs and posterior column cells yielded 16.5 × 10^6^ and 31.2 × 10^6^ read tags, respectively. We first eliminated the proprietary tag (NuGEN Technologies) from the 3′ end of each read using the FASTX-toolkit (http://hannonlab.cshl.edu/fastx_toolkit/). We then mapped 10.8 × 10^6^ (65%) and 17.1 × 10^6^ read tags (55%) of the SMNs and posterior column cells, respectively, to the mouse genome (UCSC mm9) with the transcript annotation of ENSEMBL (release e66) using TopHat^62^. Gene expression and alternative splicing events were analyzed using CuffLinks^63^ and MISO^64^, respectively.

### Staining of the tibialis anterior muscle and the diaphragm

Frozen sections of the tibialis anterior muscle were fixed with acetone for 10 min at −20 °C, washed with phosphate buffered saline (PBS) several times, and then covered with PBS containing 2% goat serum for 60 min. For staining, the sections were incubated with rabbit polyclonal anti-R-spondin 2 antibody (1:100, Abcam, ab73761), polyclonal anti-Flag antibody (1:100, Cell signaling, 2368P), or anti-GPR49 antibody for Lgr5 staining (1:100, Abcam, ab75732) overnight at 4 °C in a humidified chamber. After the removal of the primary antibody and repeated washes with PBS containing 0.05% Tween-20 (PBS-T), the sections were incubated with the goat anti-rabbit Alexa 488 secondary antibody (1:100, Molecular Probes, A21206), and Alexa594-conjugated α-bungarotoxin (1:100, Invitrogen, B13423) for 1 h. Residual antibodies were removed with repeated washes in PBS-T. Finally, the sections were coverslipped with VectaShield mounting medium containing 1.5 μg/ml 4′,6-diamidino-2-phenylindole (DAPI) (Vector Laboratories) and were visualized using an IX71 microscope (Olympus) or A1Rsi confocal microscope (Nikon).

The left diaphragm of E18.5 *Rspo2*−/− mouse was fixed with 2% paraformaldehyde in PBS for 4 h at 4 °C and was rinsed with PBS. After the removal of the connective tissue, the whole-mount diaphragm was permeabilized with 0.5% Triton X-100 in PBS for 10 min and then incubated overnight with α-bungarotoxin conjugated with the Biotin-XX Microscale Protein Labeling Kit (1:800, Invitrogen), anti-peripherin antibody (1:800, Millipore, AB1530), and anti-synaptophysin antibody (1:100, Invitrogen, 180130). After washing, the sections were incubated with Alexa 564-conjugated streptavidin (1:500, Invitrogen) or Alexa 488-conjugated anti-mouse IgG (1:500, Invitrogen). Immunostaining in the whole-mount diaphragms (*n* = 5) was quantified by two blinded observers using an FSX100 fluorescence microscope equipped with the FSX-BSX and CellSens software (Olympus). Parameters related to the NMJs and the ratio of the NMJs terminated by an axon were also quantified by two blinded observers using a confocal laser scanning microscope system (Zeiss LSM710) or FSX100 fluorescence microscope and the MetaMorph software (Molecular Devices).

### Detection of phosphorylated MuSK

To examine the effects of Rspo2 on MuSK phosphorylation, C2C12 myoblasts were seeded on a plate coated with collagen I (BD Biosciences), and were differentiated with 2% horse serum for 5 to 7 days. C2C12 myotubes were added with variable concentrations of purified agrin (R&D systems, 550-AG) and/or purified Rspo2 (R&D systems, 3266-RS) to induce MuSK phosphorylation for 30 min. C2C12 myotubes were lysed with a buffer containing 50 mM HEPES; pH 7.0, 150 mM NaCl, 10% glycerol, 1% TritonX-100, 1.5 mM MgCl_2_, 1 mM ethylene glycol tetraacetic acid (EGTA), 100 mM NaF, 10 mM sodium pyrophosphate, 1 μg/μl aprotinin, 1 μg/μl leupeptin, 1 μg/μl pepstatin A, 1 mM phenylmethanesulfonylfluoride (PMSF), 1 mM sodium orthovanadate, and x1 phosphatase inhibitor cocktail (Roche, PhosSTOP, 04906837001). Cell lysates were immunoprecipitated using 1 μg anti-MuSK antibody (C-19, Santa Cruz) and anti-Flag antibody (F1804, Sigma) attached to protein G Sepharose beads (GE Healthcare). Immunoprecipitated molecules were detected with Western blotting as described in the Materials and Methods section in the Supplementary Information.

To examine the effects of Lgr5 knockdown, L cells and C212 myoblasts were infected with a lentivirus expressing shRNA against Lgr5 (see Supplementary Information for lentivirus constructs) for 12 h. The infected L cells were transfected with Flag-tagged human *MUSK* cDNA and human *LRP4* cDNA, and cultured for 2 days. The infected C2C12 myoblasts were differentiated for 5 to 7 days. For L cells and C2C12 myotubes, shRNA against Lgr5 was induced by 2 mg/ml doxycycline (ICN Biomedicals) for 2 days, and were treated with purified recombinant Rspo2 for 30 min. Western blotting to detect phosphorylated MuSK was performed as described above.

### Co-immunoprecipitation assay

HEK293 cells were transfected with a combination of Rspo2-myc alkaline phosphatase (AP), MuSKect-mycAP, pcDNA3.1-human *LRP4*, and pcDNA3.1-human *LGR5*, and were cultured for 48 h for Fig. 3B. HEK293 cells were transfected with a combination of LRP4-Flag, pcDNA3.1 human full-length *MuSK*, and pcDNA3.1-human *LGR5* and were cultured for 48 h for Fig. 3C. HEK293 cell lysate was prepared as described above, and was immunoprecipitated using 1 μg anti-myc antibody (sc-40, Santa Cruz) or anti-Flag (F1804, Sigma) attached to protein G Sepharose beads. Immunoprecipitated molecules were detected with Western blotting as described in the Materials and Methods section in the Supplementary Information.

### Electron microscopy

At E18.5, the left diaphragm was fixed in 2% glutaraldehyde and 2% paraformaldehyde, and then was treated with 1% OsO_4_, dehydrated in ethanol, and embedded in Epon 812 (TAAB). Seven to ten continuous blocks were excised at an interval of 0.2 to 0.3 mm from the central portion of the left diaphragm. Every second block was stained for cholinesterase using the Ellman method. Only the blocks that were flanked with cholinesterase-stained blocks were used for electron microscopy. Ultrathin sections (60–70 nm) were stained with uranyl acetate and lead citrate. As the nerve terminals were immature at this stage and could not be traced to the ends where they make a synapse with a muscle fiber, we identified the NMJs by inspecting the entire ultrathin section using a JEM-1400 transmission electron microscope.

Morphometric analysis of the motor endplate was performed according to Engel and Santa^65^; the following parameters were measured: nerve terminal area in μm^2^, synaptic vesicle density in μm^2^ at the nerve terminal area, area of mitochondria/area of nerve terminal (%), the number of active zones, the diameter of synaptic vesicles, and the width of the synaptic cleft. The active zone was defined as the site of a synaptic vesicle cluster at the presynaptic membrane^59^. The postsynaptic fold was defined as the fold in postsynaptic membrane, where the fold depth was more than 70 nm and the width of fold aperture was less than a half of the fold depth. Images were quantified using the ImageJ program (http://imagej.nih.gov/ij/).

### Electrophysiology

Phrenic nerve-diaphragm preparations were obtained from 3 wild-type and 3 *Rspo2*−/− mice at E18.5. We stimulated the sciatic nerve at 2 Hz and recorded the compound muscle action potentials of the tibialis anterior muscles using a needle electrode. Miniature endplate potentials were also recorded as described previously^66^. Data were analyzed with the AxoGraph X 1.5.0 software (AxoGraph Scientific).

### Statistical analysis

Unpaired Student’s *t*-test, one-way or two-way ANOVA, and post-hoc Bonferroni adjustments were performed using Prism 5.0 (GraphPad). *P* values of 0.05 or less were considered statistically significant.

### Other materials and method

Details of other materials and methods are available in the Supplementary Information, which includes the description of the following techniques: *in situ* hybridization, staining of the spinal cord, quantitative RT-PCR, expression vectors, luciferase reporter vectors, lentiviral vectors, siRNAs, the preparation of myc-tagged proteins, cell cultures, transfections, production of conditioned medium (CM), AChR clustering assay, luciferase assay, Western blotting, the biotinylation of plasma membrane proteins, protein preparation of muscle tissues in mice.

## Additional Information

**Accession code**: Affymetrix exon array data is available under the GEO accession number GSE51122.

**How to cite this article**: Nakashima, H. *et al*. R-spondin 2 promotes acetylcholine receptor clustering at the neuromuscular junction via Lgr5. *Sci. Rep.* **6**, 28512; doi: 10.1038/srep28512 (2016).

## Supplementary Material

Supplementary Information

## Figures and Tables

**Table 1:** Parameters of the neuromuscular junction (NMJ) ultrastructure in the diaphragm of wild-type and R-spondin 2 (*Rspo2)*−/− mice at embryonic day (E) 18.5.

	Wild-type	*Rspo2*−/−	Ratio	*p*
Nerve terminal area (μm^2^)	4.17 ± 1.87 (*n* = 15)	4.81 ± 3.87 (*n* = 15)	1.15	0.185
Area of mitochondria/area of nerve terminal (%)	4.14 ± 2.60 (*n* = 15)	3.93 ± 2.47 (*n* = 15)	0.95	0.419
Diameter of synaptic vesicles (nm)	58.35 ± 16.93 (*n* = 1007)	64.10 ± 18.45 (*n* = 536)	1.10	0.00709
Density of synaptic vesicles (/μm^2^)	25.87 ± 12.37 (*n* = 15)	15.36 ± 7.53 (*n* = 15)	0.59	0.000150
Width of the synaptic cleft (μm)	146.86 ± 103.63 (*n* = 150)	209.50 ± 169.07 (*n* = 150)	1.43	2.04 × 10^−5^
Number of active zones (/synapse)	3.61 ± 2.45 (*n* = 15)	1.72 ± 1.64 (*n* = 15)	0.48	1.34 × 10^−5^
Number of postsynaptic folds (/synapse)	1.20 ± 0.86 (*n* = 15)	0.20 ± 0.41 (*n* = 15)	0.17	0.000367

Blinded morphometric analysis was performed on electron microscopic images of the NMJs in the diaphragms at E18.5. Four wild-type and four *Rspo2*−/− mice were analyzed. Mean and the standard deviation are indicated. Ratio is calculated by dividing the respective mean value in *Rspo2*−/− mice by that in wild-type mice. Statistical significance was calculated with the Student’s *t*-test.

**Table 2:** Microelectrode measurements and repetitive nerve stimulation in R-spondin 2 (*Rspo2)*−/− mice at embryonic day (E) 18.5.

	Wild-type	*Rspo2*−/−	*p*
MEPP amplitude (mV)	3.38 ± 0.18 (*n* = 22)	3.86 ± 0.70 (*n* = 25)	0.04
MEPP frequency (sec^−1^)	0.65 ± 1.10 (*n* = 22)	0.07 ± 0.02 (*n* = 25)	0.01
CMAP area (%)	102.2 ± 1.6 (*n* = 4)	92.5 ± 0.7 (*n* = 4)	0.0031

Miniature endplate potentials (MEPPs) were recorded from the left diaphragm of wild-type and *Rspo2*−/− mice at E18.5. Relative areas of the fifth and first compound muscle action potentials (CMAPs) at the 2-Hz stimulation of the sciatic nerve are indicated. Statistical significance (*p*) was calculated with the *t*-test. Mean and standard error of mean (SEM) are indicated.

**Table 3:** Neuromuscular junction (NMJ) phenotypes of R-spondin (*Rspo2)*, agrin (*Agrn)*, wnt4 (*Wnt4*), and β-catenin (*Ctnnb1)* deficient mice.

Knocked out gene	*Rspo2*	*Agrn*^54^	*Wnt4*^15^	*Ctnnb1*^18^
AChR clusters				
Size	↑	↓	↑	↑
Band width of AChR clusters	↑	↑	↑	↑
Basal lamina	Normal	Patchy	n.a.	Normal
Synapses				
Synaptic cleft	Widened	Widened	n.a.	Normal
Postsynaptic fold	↓	↓	n.a.	n.a.
Motor axons				
Terminal arborization	Increased branches	Increased branches	Increased branches	Decreased branches
Nerve terminals				
Number of active zones	↓	↓	n.a.	↓
Number of synaptic vesicles	↓	↓	n.a.	↓

n.a., not available.
