# Supplementary material for: R-spondin 2 promotes acetylcholine receptor clustering at the neuromuscular junction via Lgr5
Source: Sci Rep. 2016 Jun 22;6:28512. doi: 10.1038/srep28512 (PMC4916433; doi:10.1038/srep28512)

R-spondin 2 promotes acetylcholine receptor clustering at the neuromuscular junction via Lgr5

Hiroaki Nakashima[1,2,*], Bisei Ohkawara[1,*], Shinsuke Ishigaki[3], Takayasu Fukudome[4], Kenyu Ito[1,2], Mikito Tsushima[1,2], Hiroyuki Konishi[5], Tatsuya Okuno[1], Toshiro Yoshimura[6], Mikako Ito[1], Akio Masuda[1], Gen Sobue[3], Hiroshi Kiyama[5], Naoki Ishiguro[2], Kinji Ohno[1]

[1]Division of Neurogenetics, Center for Neurological Diseases and Cancer, Nagoya University Graduate School of Medicine, Nagoya, Japan

[2]Department of Orthopedic Surgery, Nagoya University Graduate School of Medicine, Nagoya, Japan

[3]Department of Neurology, Nagoya University Graduate School of Medicine, Nagoya, Japan

[4]Department of Neurology, Nagasaki Kawatana Medical Center, Nagasaki, Japan,

[5]Department of Functional Anatomy and Neuroscience, Nagoya University Graduate School of Medicine, Nagoya, Japan

[6]Department of Occupational Therapy, Nagasaki University School of Health Sciences, Nagasaki, Japan

*These authors contributed equally to this work.

Supplemental Information

Materials and methods

***In situ* hybridization**

Sections (18 µm) of the lumbar spinal cord of six-week-old C57BL/6J mouse were immediately fixed by 4% paraformaldehyde in DEPC-treated phosphate-buffered saline (PBS) for 20 min. The sections were incubated twice for 15 min in PBS with 0.1% DEPC, and equilibrated for 15 min in DEPC-treated 5 x SSC. The sections were then prehybridized for 2 h at 58˚C, in the hybridization mixture (50% formamide, 5 x SSC, and 40 µg/ml salmon sperm DNA). Mouse *Rspo2* cDNA spanning positions 1972 and 2539 was cloned into pGEM-T Easy vector (Promega). Digoxigenin-labeled antisense *Rspo2* RNA probe was synthesized using Roche DIG system. The probe was denatured for 5 min at 80˚C and added to the hybridization mixture to a final concentration of 400 ng/ml. Each section was incubated with 20 µl of the hybridization mixture at 58˚C for 12 h. After the incubation, the sections were washed for 30 min in 2 x SSC at room temperature, 1 h in 2×SSC at 65˚C, and 1 h in 0.1×SSC at 65˚C. The sections were then equilibrated for 5 min in Buffer 1 (100 mM Tris-HCl pH 7.5 and 150 mM NaCl) and incubated for 2 h at room temperature with alkaline phosphatase-coupled anti-digoxigenin antibody (1:2000) in Buffer 1 containing 0.5% Blocking Reagent (Roche, cat. no. 1096176). The sections were washed twice for 15 min in Buffer 1, and were equilibrated for 5 min in Buffer 2 (Buffer 1 and 0.5% Blocking Reagent). The sections were incubated overnight in Buffer 3 (100 mM Tris-HCl pH 9.5, 5 M NaCl, and 1 M $MgCl_2$) with the NBT/BCIP Stock Solution (Roche), followed by washing with PBS. Microscopic images were analyzed with a SZX16 stereomicroscope (Olympus) fitted with a DP-70 camera (Olympus).

Quantitative RT-PCR of the spinal cord and the diaphragm muscle

Spinal cords and diaphragm muscles were harvested from E13.5, E18.5, and adult mice.

Total RNA was extracted using Trizol reagent (Invitrogen), and was reverse-transcribed by Oligo $(dT)_{20}$ Primer (Life Technologies) using ReverTra Ace reverse-transcriptase (Toyobo). cDNA levels were analyzed by qRT-PCR in triplicate using SYBR Premix Ex Taq (Takara) on LightCycler 480 (Roche). cDNA levels were normalized to *Gapdh*. The primer sequences are listed in Supplementary Table S2.

Staining of the spinal cord

The spinal cord of adult or E18.5 mice with vertebral bones were fixed with 4% parafolmaldehyde at 4°C overnight, washed with PBS several times, removed with the bone tissues, and then treated with 10%, 15%, and 30% sucrose in PBS sequentially until the tissues sink to the bottom of a vial. Frozen sections of the spinal cord were fixed with acetone for 5 min on ice, washed with PBS several times, covered with Image-iT FX Signal Enhancer (Cell Signaling, #11932) for 30 min and then covered with PBS containing 5% horse serum for 30 min. For staining, the sections were incubated with rabbit anti-Islet1/2 antibody (1:100, Santa Cruz, sc30200), rabbit polyclonal anti-R-spondin 2 antibody (1:500, Abcam, ab73761), or goat polyclonal anti-choline acetyltransferase (ChAT) antibody (1:100, Millipore, AB143) overnight at 4°C in a humidified chamber. After repeated washes with PBS, sections of the E18.5 spinal cord stained for Islet1/2 were incubated with the goat anti-rabbit Alexa 488 secondary antibody (1:100, Molecular Probes, A21206). Sections of the adult spinal cord double-stained for Rspo2 and ChAT were incubated with biotinylated donkey anti-goat secondary antibody for ChAT (1:300, Vector Laboratory, BA9500) for 30 min, and then treated with a combination of the goat anti-rabbit Alexa 488 secondary antibody for Rspo2 (1:100, Molecular Probes, A21206) and streptavidin-conjugated Alexa 594 (1:1000, Invitrogen, S11227) for detecting ChAT for 1 h. Residual antibodies were removed with repeated washes in PBS-T. Finally, the sections were coverslipped with VectaShield mounting medium containing 1.5 µg/ml 4',6-diamidino-2-phenylindole (DAPI) (Vector Laboratories) and were visualized using an IX71 microscope (Olympus) or A1Rsi confocal microscope (Nikon). The Islet1/2-immunopositive SMNs were counted by two blinded observers and averaged.

AChR clustering assays

C2C12 myoblasts were seeded on a plate coated with collagen I (BD Biosciences) and treated with the lentivirus for 12 h. After differentiation, C2C12 myotubes were treated with doxycycline for 2 d to induce shRNA expression and then treated with Rspo2 protein, agrin protein or Rspo2-CM to induce AChR clusters for 12 h. Thirty min before fixation in 2% paraformaldehyde, the cells were incubated with 10 μg/ml Alexa594-conjugated α-bungarotoxin (Invitrogen) for 30 min to label AChR. Fluorescent images were observed under an Olympus XL71 fluorescence microscope and analyzed with MetaMorph software (Molecular Devices). The lengths of AChR clusters and myotubes were defined as the longest axes of Alexa594 signals and GFP signals, respectively, in the lentivirus-transfected cells. AChR clusters with an axis length of less than 4 μm were excluded from the total count of AChR clusters.

Expression vectors, luciferase reporter vectors, lentiviral vectors, and siRNAs

The full-length human *LRP4* cDNA (Open Biosystems) was cloned into the EcoRI site and the full-length human and mouse *LGR5* cDNA (Open Biosystems) was cloned into BamHI and NotI sites of the pcDNA3.1 mammalian expression vector (Invitrogen). The mouse *Musk* cDNA in pExpress-1 was purchased from Open Biosystems. ATF2-Luc to quantify the JNK signaling activity[1] and phRL-TK Renilla luciferase vector (Promega) were used for the luciferase reporter assay. The human full-length *MUSK* and full-length *LRP4* cDNA was subcloned into EcoRI and XbaI sites or EcoRI sites downstream of 3xFlag epitope of a mammalian expression vector p3xFlag-CMV-14 (Sigma Aldrich). For lentivirus vector expressing shRNA against *Lgr5*, double stranded oligonucleotides (sense, 5’-gatcccggttagagttttaagaaaattcaagagattttcttaaaactctaacctttttggaaa-3’ and antisense, 5’-agcttttccaaaaaggttagagttttaagaaaatctcttgaattttcttaaaactctaaccgg-3’ for shLgr5-3; sense, 5’-gatcccggccgtctgtgatcagttattcaagagataactgatcacagacggcctttttggaaa-3’ and antisense, 5’-agcttttccaaaaaggccgtctgtgatcagttatctcttgaataactgatcacagacggccgg-3’ for shLgr5-5, where the complementary nucleotides are underlined) were cloned into a lentiviral vector pLenti CMV GFP x2

DEST, which was kindly provided by Dr. Eric Campeau at the University of Massachusetts Medical School. For efficient secretion of Rspo2 into the culture medium, the human C-terminal-deleted *RSPO2* cDNA (amino acids 1-218) was cloned into APtag-5 (GenHunter) at the HindIII and SnaBI sites (Rspo2-mycAP). The clone carried the Igk-originated signal peptide upstream of the insert and the myc-tag/alkaline phosphatase downstream of the insert. Lack of PCR artifacts was verified by sequencing the entire inserts for all the clones. The extracellular domain of mouse Musk cDNA fused to a myc-tag and alkaline phosphatase (MuSKect-mycAP) was kindly provided by Dr. Lin Mei. The double stranded siRNA against human *LGR5* were: sense, 5'-gaaagaugcuggaauguuutt-3' and antisense, 5'-aaacauuccagcaucuuuctt-3' for siLgr5-1; sense, 5'-gaacuaggauuucauagcatt-3' and antisense, 5'-ugcuaugaaauccuaguuctt-3' for siLgr5-2.

Cell cultures, transfections and preparation of conditioned medium (CM)

HEK293, L, and C2C12 cells were cultured in the Dulbecco's Modified Eagle's medium (DMEM) supplemented with 10% fetal bovine serum (FBS). Transfection was performed with Lipofectamine 2000 (Invitrogen) according to the manufacture's protocols. Lentivirus expressing shRNA was prepared as described by Campeau and colleagues[2]. Briefly, HEK293 cells were plated in a 150-mm dish on the day before transfection. We introduced pLP1, pLP2, and pLP/VSVG plasmids (ViraPower Packaging Mix, Invitrogen) as well as pLenti vector into HEK293 cells with Lipofectamine 2000 according to the manufacturer's protocols. At 48 h and 96 h after transfection, we filtered the media containing the virus particles using the Millex-HV 0.45 μm PVDF filter (Millex). Viruses were concentrated in a Beckman SW28 rotor at 21,000 rpm for 2 h at 4˚C and resuspended in 4 ml of Hank's Buffered Saline solution (HBS, Invitrogen). After the second ultracentrifugation in a Beckman 55Ti rotor at 21,000 rpm for 90 min, the viral pellet was resuspended in 100 μl of HBS. The lentivirus was added to the medium of L cells or C2C12 cells. After 48 h, we confirmed that more than 90% of cells were positive for GFP signals driven by CMV in pLenti. HEK293 cells were transfected with pEGFP-N1 (Clontech) to make control-CM or Rspo2-mycAP to make Rspo2-CM. The cells were cultured in 10% FBS/DMEM for L cells or

HEK293 cells, or in 2% horse serum/DMEM for C2C12 cells. The CM was harvested at 48 and 96 h after the transfection. Recombinant rat C-terminal agrin and recombinant Rspo2 were purchased from R&D systems.

Luciferase assays

HEK293 cells were transfected with ATF2-Luc and phRL-TK along with the *Musk* and *Lrp4* cDNAs. Cells were cultured for 24 h in the presence or absence of purified Rspo2 (100ng/ml) or/and agrin protein (10 ng/ml) in a 96-well plate. Cells were lysed with the passive lysis buffer (Promega) and assayed for the luciferase activity using the Dual luciferase system (Promega). Each experiment was done in triplicate.

Western blotting

Total or precipitated proteins were dissolved in 1x Laemmli buffer, separated on a 12.5%, 10% or 7.5% SDS-polyacrylamide gel, and transferred to a polyvinylidene fluoride membrane (Immobilon-P, Millipore). Membranes were washed in Tris-buffered saline containing 0.05% Tween 20 (TBS-T) and blocked for 1 h at room temperature in TBS-T with 3% bovine serum albumin. The membranes were incubated overnight at 4˚C either with the mouse monoclonal anti-Flag M2 (Sigma-Aldrich, dilution 1:4000), anti-β-actin (Santa Cruz Biotechnology, sc-47778, dilution 1:200), anti-myc (Santa Cruz, sc-40, 1:1000), the rabbit polyclonal anti-Lgr5 (anti-GPCR GPR49, abcam, ab75732, 1:1000), anti-Transferrin (abcam, ab84036, 1:1000), goat polyclonal anti-MuSK (R&D, AF562, 1:1000), anti-phosphotyrosine antibody (4G10, Upstate, 1:1000), anti-Flag (Sigma, F1804, 1:4000), anti-rapsyn (abcam, ab156002, 1:1000) or anti-LRP4 (abcam, ab85697, 1:1000) antibody. The membranes were washed three times for 10 min with TBS-T and incubated with a secondary goat anti-mouse IgG antibody (GE Healthcare, NA931V, 1:6000), donkey anti-rabbit IgG antibody (GE Healthcare, NA9340V, 1:6000), or mouse anti-goat IgG (Santa Cruz Biotechnology, sc-2345, 1:6000 conjugated to horseradish peroxidase (HRP) for 1 h at room temperature. The blots were detected with the Amersham ECL Western blotting detection reagent (GE Healthcare) and quantified

with the ImageJ program (http://imagej.nih.gov/ij/).

Biotinylation of proteins on plasma membrane

C2C12 cells were cultured with or without recombinant Rspo2 protein (100 ng/ml, R&D systems) for 48 h. The cells were washed twice with PBS containing 1 mM $MgCl_2$ and 0.1 mM $CaCl_2$ (PBS/CM), followed by incubation with 0.5 mg/ml sulfo-NHS-SS-biotin (Pierce) in PBS/CM on ice for 30 min. The cells were then washed once with PBS/CM and incubated with 10 mM monoethanolamine for quenching free biotin. The cells were harvested with RIPA buffer (Pierce) after several washing with ice-cold PBS and the cell lysates were incubated with streptavidin sepharose beads (GE healthcare) to purify the biotinylated cell membrane proteins. Western blotting was performed as described above.

Protein preparation of muscle tissues in mice

The left diaphragm muscle in each mouse was dissected at E18.5 and crushed using the Multi-Beads Shocker (Yasui Kikai Corp.). The Minute Plasma Membrane isolation kit (Invent Biotechnologies) was used to isolate total and plasma membrane protein fractions from the crushed muscles. All procedures were performed on ice and followed the manufacturer’s protocols. Western blotting was performed as described above.

References

1. van der Sanden, M. H., Meems, H., Houweling, M., Helms, J. B. & Vaandrager, A. B. Induction of CCAAT/enhancer-binding protein (C/EBP)-homologous protein/growth arrest and DNA damage-inducible protein 153 expression during inhibition of phosphatidylcholine synthesis is mediated via activation of a C/EBP-activating transcription factor-responsive element. *J Biol Chem* **279**, 52007-52015 (2004).
2. Campeau, E. *et al.* A versatile viral system for expression and depletion of proteins in

mammalian cells. *PLoS One* **4**, e6529 (2009).

Legends for Supplementary Figures

Supplementary Figure S1. Expression and localization of Rspo2 in the spinal cord and the muscle tissue

(A) mRNAs expressed in SMNs and the posterior horn cells were analyzed by RNA-seq. Gene expression levels are indicated by the fragments per kilobase of exon per million mapped fragments (FPKM) generated by Cufflinks.

(B) Immunostaining for Rspo2 expressed in SMNs of adult spinal cord (green; upper image). Region in a white square is shown in lower panels. Rspo2 and choline acetyltransferase (ChAT) are shown in green and red, respectively.

(C) Localizations of Rspo2 and AChR in a cross section of tibialis anterior muscle of wild-type (+/+) and *Rspo2*-deficient (-/-) mice at embryonic day (E) 18.5. AChR was stained with Alexa 594-conjugated α-bungarotoxin (red). Rspo2 was stained with anti-Rspo2 IgG (green). Anti-Flag IgG was used as a control. Arrows point to AChR clusters.

Supplementary Figure S2. Rspo2 conditioned medium (CM) and purified Rspo2 protein induce AChR clustering and phosphorylation of MuSK

(A) Quantification of the additive effect of agrin and Rspo2 on AChR clustering and myotube formation shown in Fig. 2C. Mean and SD are indicated (**$p < 0.01$ using the t-test, $n = 3$).

(B) Quantification of the additive effect of agrin and Rspo2 on MuSK phosphorylation shown in Fig. 2G. Values are normalized by that with 0.1 nM agrin. Mean and SD (**$p < 0.01$ using the t-test, $n = 3$) are indicated.

(C) Quantification of the additive effect of agrin and Rspo2 on rapsyn expression shown in Fig. 2H. Values are normalized by that with 0.1 nM agrin. Mean and SD (**$p < 0.01$ using the t-test, $n = 3$) are indicated.

(D) Instead of using purified recombinant Rspo2 in Fig. 3G, we used Rspo2-containing

conditioned medium (CM) and the other experimental conditions are identical to those of Fig. 3G. Mean and SD are indicated (**$p < 0.01$ by t-test, $n = 3$).

Supplementary Figure S3. Lgr5 and Wnt ligands are required for Rspo2-induced MuSK phosphorylation

(A) Quantification of co-immunoprecipitated Lgr5 shown in Fig. 3B. Values are normalized to that of Rspo2. Mean and SD are indicated (**$p < 0.01$ by t-test, $n = 3$).

(B) Quantification of co-immunoprecipitated Lgr5 shown in Fig. 3C. Values are normalized to that of Lgr5. Mean and SD are indicated (**$p < 0.01$ by t-test, $n = 3$).

(C) RT-PCR to quantify the effects of siLgr5-1 and -2 on *LGR5* expression in HEK293 cells. Mean and SD are indicated (**$p < 0.01$ by t-test, $n = 3$).

(D) Rescue experiments of Fig. 3C. HEK293 cells were transfected with ATF2-luciferase reporter, siRNA (siControl, siLgr5-1, or siLgr5-2), and mouse full-length *Lgr5* cDNA. Rspo2 (100 ng/ml) or BSA (100 ng/ml) was added to the medium and the relative luciferase activities (RLA) were measured at 48 h. RLA are normalized to that with BSA with siControl. Mean and SD are indicated (**$p < 0.01$ using the t-test, $n = 3$). Efficiencies of siLgr5-1 and siLgr5-2 are indicated in **B**.

(E) RT-PCR to quantify the effects of lentivirus expressing shLgr5-3 and -5 on *Lgr5* expression in mouse fibroblast L cells. Mean and SD are indicated (**$p < 0.01$ by t-test, $n = 3$).

(F) Quantification of phosphorylated MuSK induced by up- and down-regulation of Lgr5 shown in Fig. 3D. The ratio was normalized to that of shControl. Mean and SD (**$p < 0.01$ by t-test, $n = 3$) are indicated.

(G) Western blotting to quantify the effects of lentivirus expressing shLgr5-3 and -5 on Lgr5 protein expression in C2C12 myotubes. Lower panel shows quantification of Lgr5 protein levels. The ratio was normalized to that with shControl. Mean and SD are indicated (**$p < 0.01$ by t-test, $n = 3$).

(H) Quantification of the effect of Lgr5 knockdown on phosphorylated MuSK in agrin- or Rspo2-stimulated C2C12 myotubes shown in Fig.3E. The ratio was normalized to that of cells treated with 0.1 nM agrin and shControl.Mean and SD (**$p < 0.01$ by *t*-test, $n = 3$) are indicated.

(I) IWP-2 (2 μM) abrogates Rspo2-mediated MuSK phosphorylation in C2C12 myotubes. C2C12 myotubes were treated with 0.1 nM agrin or 0.1 nM Rspo2. Phosphorylated MuSK is detected as in Fig. 2E. The ratio of phosphorylated and total MuSK was normalized to that with 0.1 nM agrin. Mean and SD (**$p < 0.01$ by *t*-test, $n = 3$) are indicated.

Supplementary Figure S4. Membrane-bound LRP4 is stabilized in *Rspo2*-/- mice

(A-C) Western blotting of the indicated fractions of diaphragm muscles in wild-type (+/+), heterozygous *Rspo2*-knockout (+/-), and homozygous *Rspo2*-knockout (-/-) mice **(A)**. Intensities of membrane-bound LRP4 **(B)** and MuSK **(C)** were normalized to that of transferrin receptor, and also to the ratio in wild-type mice (+/+). Mean and SD (**$p < 0.01$ by *t*-test, $n = 3$) are indicated.

(D, E) Western blotting of C2C12 myotubes treated with purified Rspo2 (0.1 nM) for 48 h **(D)**. After the incubation, membrane proteins are biotinylated and precipitated with streptavidin. **(E)** The ratio of plasma membrane to total LRP4, MuSK, Lgr5 and transferrin receptor proteins are normalized to those with BSA-treated control (BSA). Mean and SD (**$p < 0.01$ by *t*-test, $n = 3$) are indicated. The mean values are also shown in **D**.

Supplementary Figure S5. NMJs in diaphragm of Rspo2-/- at E14.5 and E18.5 were innervated with neuronal axons.

(A, B) Collapsed z-serial high-power field confocal images of the whole-mount staining of the left diaphragms **(A)** and blinded morphometric analysis of AChR clusters **(B)** of wild-type and *Rspo2*-/- mice at E14.5. AChR is stained with Alexa546-conjugated α-bungarotoxin in red. Phrenic nerves and terminals are immunostained with anti-peripherin and

anti-synaptophysin antibodies, respectively, both in green. The intensity indicates the sum of intensity of AChRs divided by the number of NMJs. Perimeter indicates the circumference of continuous AChR clusters. Length is the longest axis of AChR cluster. Mean and SD (n = 6) are indicated. ***p < 0.005; *p < 0.05; and n.s., not significant by *t*-test.

(C-E) Representative left diaphragms of wild-type and *Rspo2*-/- at E18.5 **(C)** stained for phrenic nerve branches and terminals with anti-peripherin and anti-synaptophysin antibodies, respectively. An arrow and an arrowhead point to representative second and third branches, respectively. Blinded quantification of the length **(D)** and the number **(E)** of axonal branches in embryonic development.

(F, G) Surface views of the left diaphragms isolated from wild-type and *Rspo2*-/- mice at E18.5. AChR and neuronal axons were stained with Alexa546-conjugated α-bungarotoxin (red) and anti-peripherin antibody (green), respectively. The AChR clustering in *Rspo2*-/- diaphragms were disseminated but innervated with motor neurons. **G** shows blinded quantification of the ratio of the NMJs terminated by an axon relative to total NMJs. Mean and SD (n = 6) are indicated. n.s., not significant by *t-test*.

Supplementary Figure S6. NMJs in diaphragm of Rspo2-/- at E18.5 is compromised.

(A) Low magnification images of the electron micrographs in Fig. 5J and K (indicated by red boxes). SV, synaptic vesicles; TS, terminal Schwann cell.

(B) Additional representative electron micrographs, other than Fig. 5J and K, of the neuromuscular junctions (NMJs) in the diaphragm of wild-type and *Rspo2-/-* mice at E18.5. The red two-headed arrow indicates a widened synaptic cleft in *Rspo2-/-* mice. The closed arrowhead at wild-type endplate points to a postsynaptic fold. Blinded morphometric measurements are shown in Table 1. High magnifications of red boxes are shown in lower panels. Note bigger but fewer synaptic vesicles (red arrows) in *Rspo2*-/- mice. SV, synaptic vesicles.

Supplemental Tables

Table S1. Mouse SMN-specific genes identified by laser capture microdissection followed by microarray analysis

Fold-change	High in SMNs	Low in SMNs	Total
≤ 2	4,430	3,270	7,700
> 2	2,014	3,006	5,020
> 10	164	286	450

Gene expression levels are compared between SMNs and posterior horn cells using the core dataset of Affymetrix Exon 1.0 ST array. Only genes with $p < 0.05$ by *t*-test ($n = 3$) are indicated. Fold-change is a difference between the signal intensity of a gene in SMNs and that in poster horn cells. Fold-change is calculated to be no less than 1.0. The numbers of genes with the indicated fold-change value are shown. The array data is deposited in the Gene Expression Omnibus (GEO) database with the accession number of GSE51122.

Table S2. Primer sequences for quantitative RT-PCR

Target genes	Forward primers	Reverse primers
mouse *Rspo2*	5’-ACCGATGGAGACGCAATAAG-3’	5’-CATCTGGACATTCATCAAAGCAGC-3’
mouse *Lgr5*	5’-GAGCGTTCGTAGGCAACCCTTCTC-3’	5’-GGTGGCAGTTCCTGTCAAGTGAGG-3’
human *LGR5*	5’-ATCTCATCTCTTCCTCAAA-3’	5’-CTTCTAATAGGTTGTAAGACA-3’
mouse *Gapdh*	5’-ACCCCTTCATTGACCTCAAC-3’	5’-TCCCGTTGATGACAAGCTTC-3’
human *GAPDH*	5’-TGCACCACCAACTGCTTAGC-3’	5’-GGCATGGACTGTGGTCATGAG-3’

Supplementary Figure S1

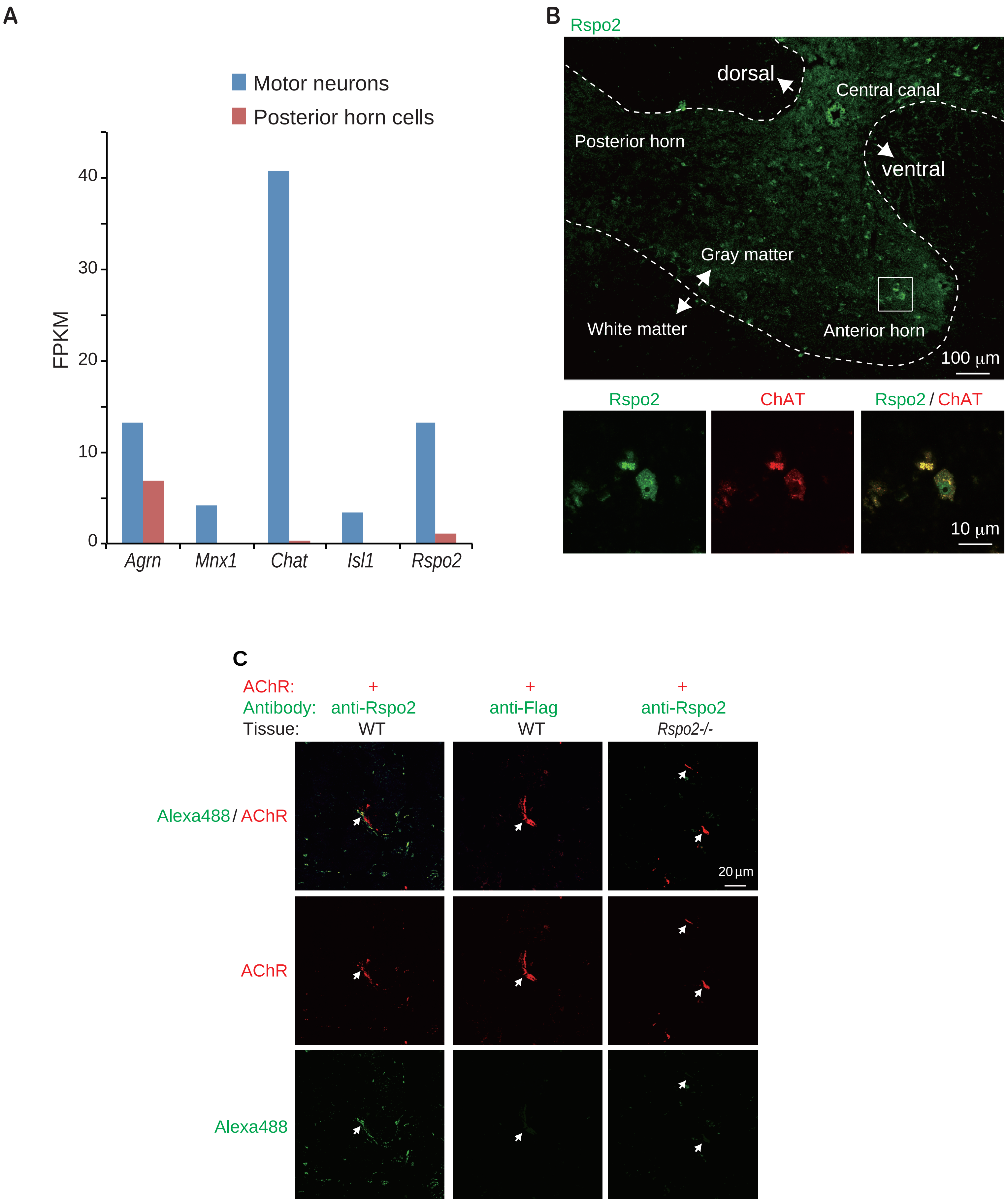

Supplementary Figure S2

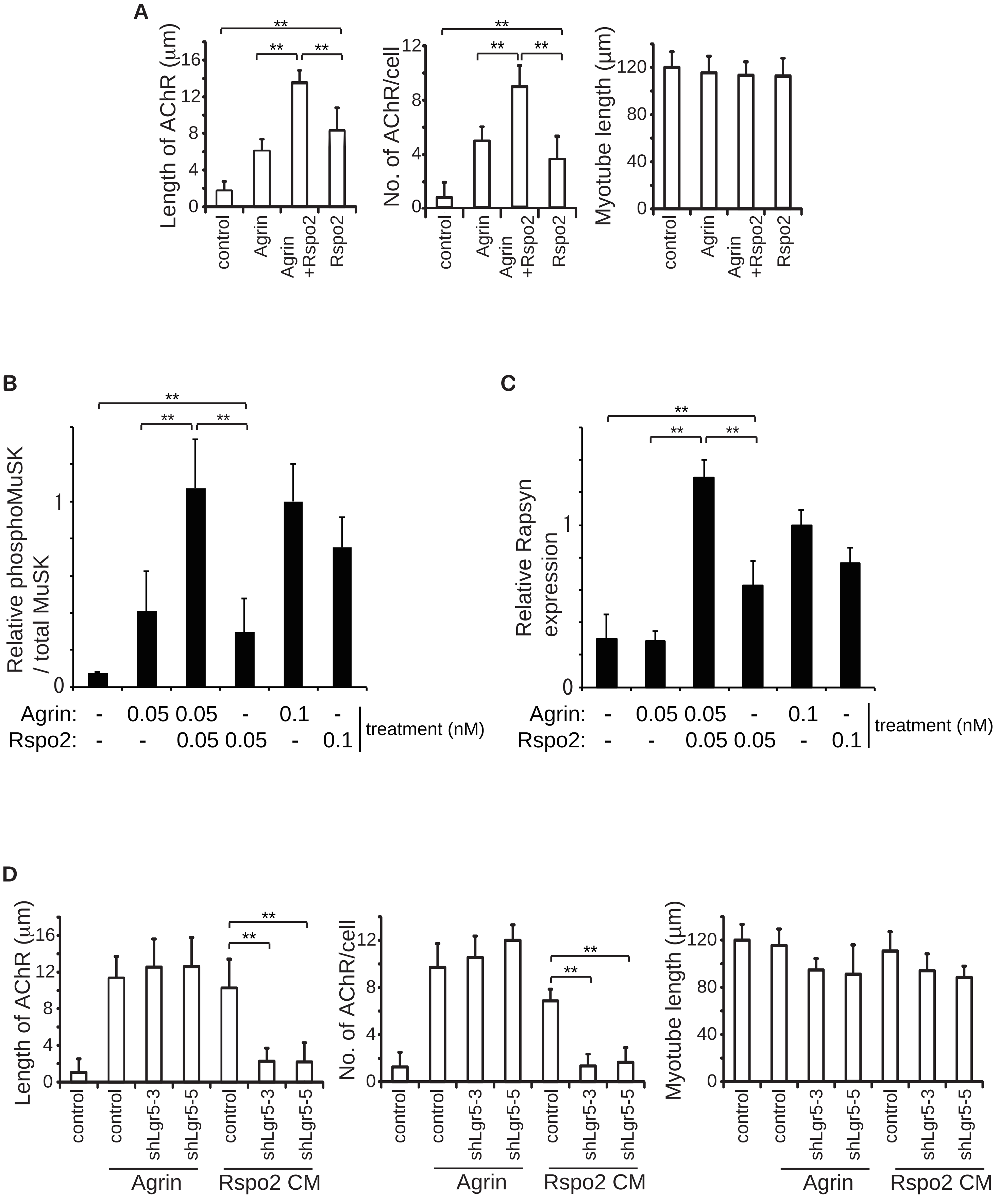

Supplementary Figure S3

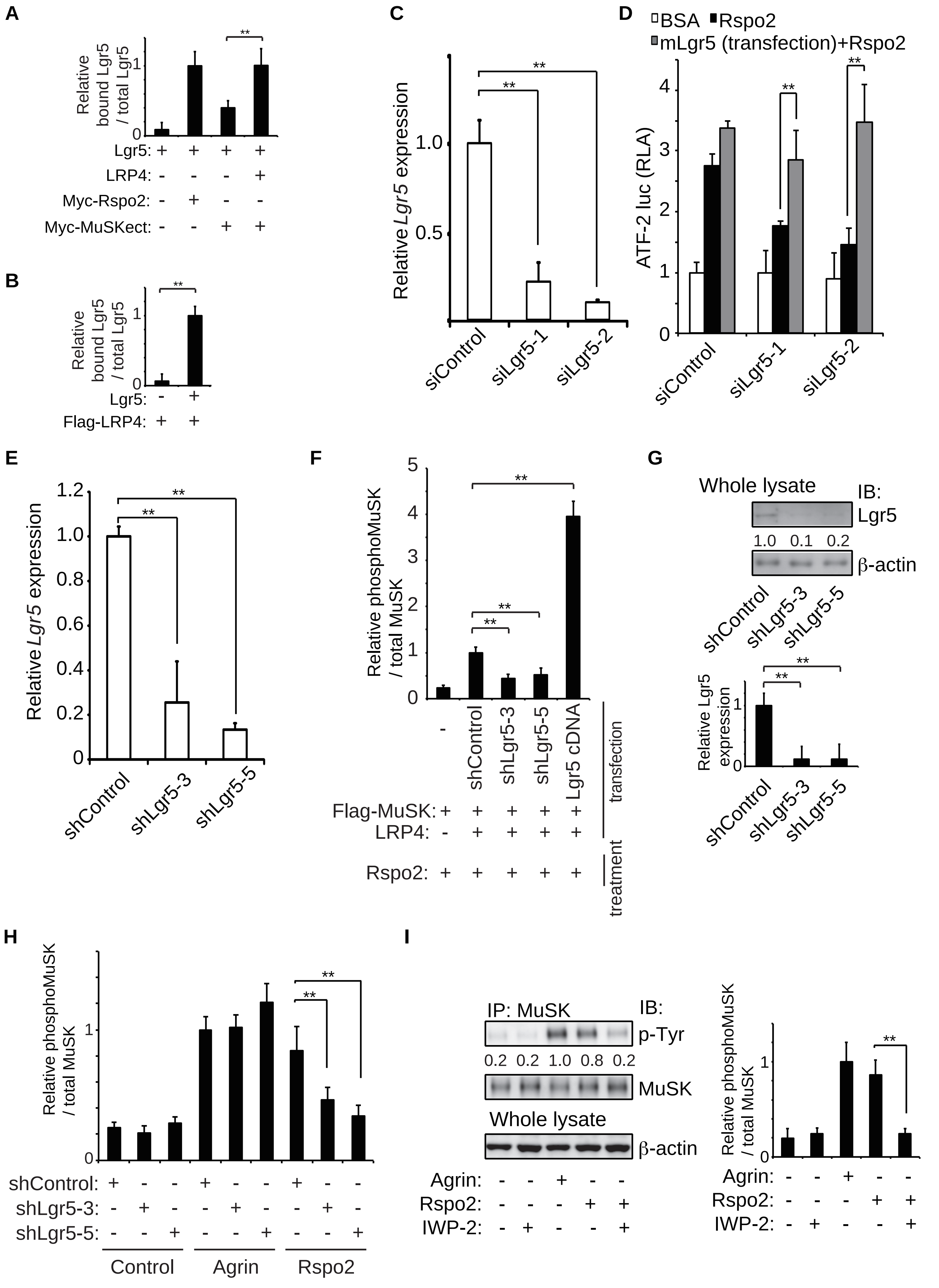

Supplementary Figure S4

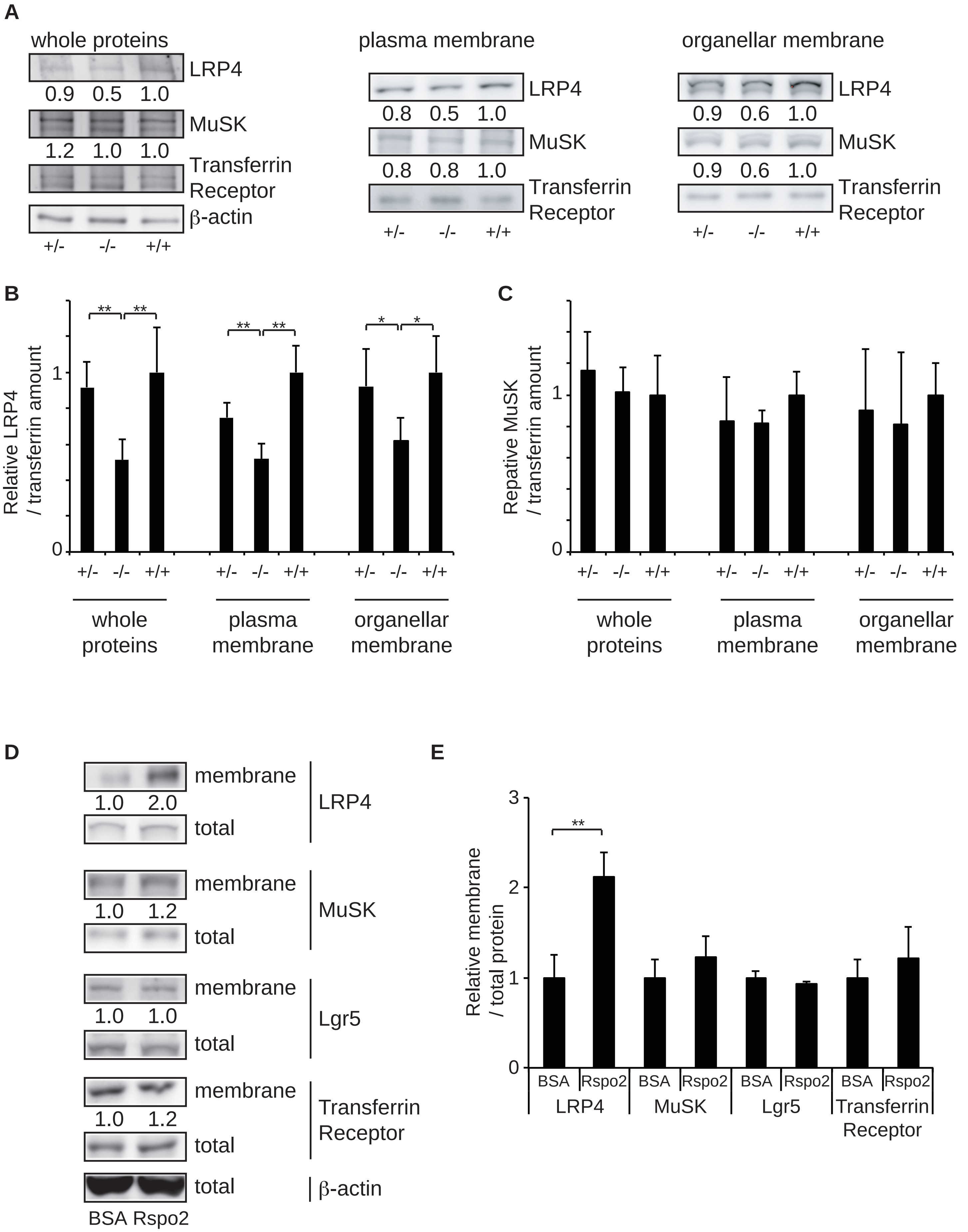

Supplementary Figure S5

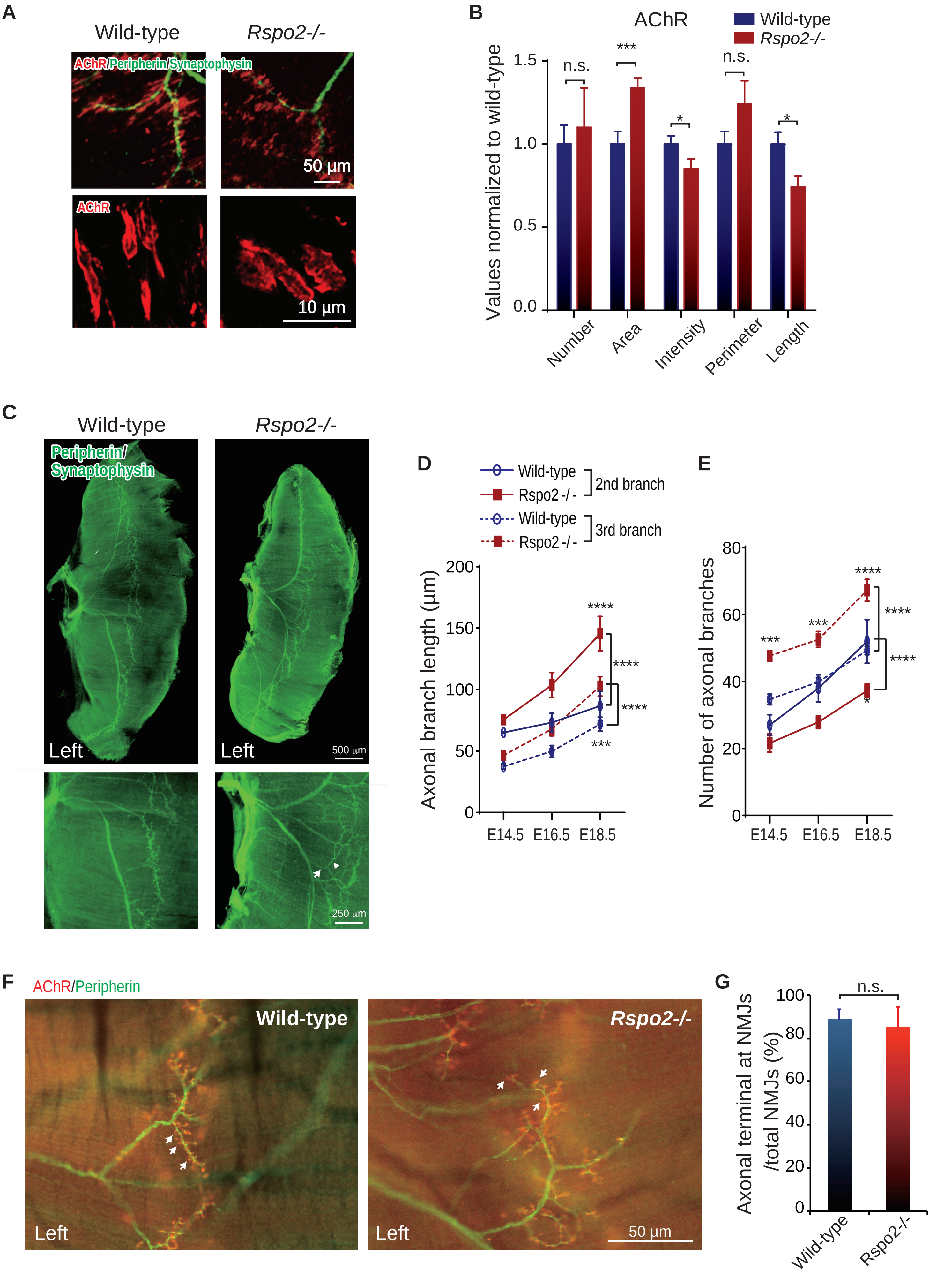

Supplementary Figure S6

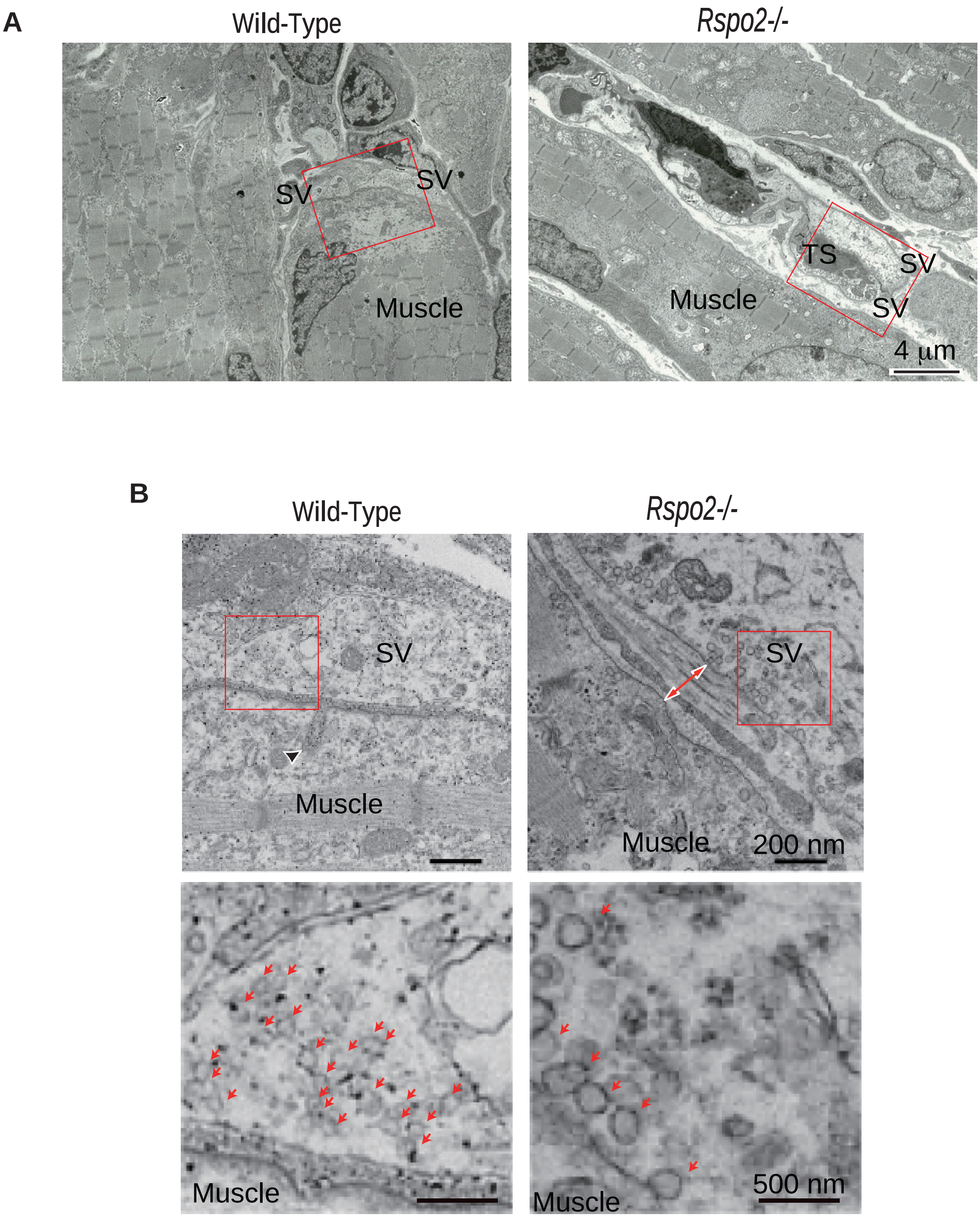

Supplement: Supplementary Information [file srep28512-s1.pdf]
